# Responsible Stimulus Selection in Neuromarketing: A Critical Narrative Review and Normative Framework for Ethical, Sustainable, and Replicable Consumer Research

**DOI:** 10.3390/bs16071115

**Published:** 2026-07-03

**Authors:** Alberto Ruiz-Osta, Casandra I. Montoro, Eduard Cristobal-Fransi

**Affiliations:** 1 Department of Business and Economics, Faculty of Law, Economics and Tourism, University of Lleida, 25001 Lleida, Spain; eduard.cristobal@udl.cat; 2 Department of Psychology, University of Jaén, 23071 Jaén, Spain; imontoro@ujaen.es

**Keywords:** neuromarketing, ethical, sustainable, replicable, consumer research, affective norms, emotions

## Abstract

Neuromarketing has emerged as a prominent methodological approach for investigating the implicit cognitive and affective processes underlying consumer decision making. By employing neuroscientific and psychophysiological techniques, it enables researchers to move beyond self-report measures and capture responses that consumers cannot always articulate explicitly. Despite these advances, a fundamental component of experimental design—the selection of affective stimuli—remains conceptually underexamined within neuromarketing research. This article adopts a structured narrative review to examine how affective stimuli are selected, documented, and justified in neuromarketing research. It develops a conceptual and normative framework that reconceptualizes stimulus selection as a decision with ethical, scientific, and sustainability implications rather than a purely technical methodological choice. The review critically examines the widespread reliance on ad hoc stimuli, discusses the potential and limitations of standardized affective databases and related resources, and highlights the need for marketing-specific stimulus repositories. By reframing stimulus selection as a core component of responsible research practice, this study contributes to emerging debates on responsible neuromarketing and provides guidance for more transparent, replicable, ethical, and sustainable neuromarketing research in academic and applied contexts.

## 1. Introduction

The increasing complexity of contemporary markets has intensified academic attention to the role of responsibility in marketing practice. Recent research highlights the need for strategies that are not only effective but also ethical, transparent, and aligned with broader societal objectives, such as consumer well-being and sustainability (Christensen et al., 2022; Vredenburg et al., 2020). This concern is particularly salient in an increasingly immersive digital ecosystem, in which technologies such as virtual reality (VR), augmented reality (AR), and metaverse environments are transforming the ways consumers interact with brands, products, and commercial experiences. Unlike traditional digital channels, these technologies enable the creation of highly realistic, interactive, and emotionally engaging stimuli capable of modulating consumer perception, attention, psychological presence, and affective responses. In this context, understanding the psychological mechanisms underlying consumer decision-making is essential for designing marketing interventions that are not only effective, but also more responsible, conscious, and oriented towards well-being (Dwivedi et al., 2023).

However, numerous studies have shown that many factors influencing consumer behavior operate at implicit levels and are not always accessible through traditional self-report measures (Greenwald & Banaji, 1995; Nisbett & Wilson, 1977). Consumers may be unable—or unwilling—to fully articulate their motivations or emotions due to introspective limitations and social desirability bias. Consequently, approaches based solely on surveys provide only a partial view of the cognitive and affective processes that shape consumption decisions (Ackermann & Mathieu, 2015).

Neuromarketing has emerged as a promising approach to address these limitations. By integrating neuroscientific and psychophysiological techniques—such as functional magnetic resonance imaging (fMRI), electroencephalography (EEG), event-related potentials (ERP), electrocardiography (ECG), eye-tracking, and electrodermal activity (EDA)—it enables the capture of indicators of attention, affective responses, and motivational states that are less dependent on conscious introspection (Ariely & Berns, 2010; Plassmann et al., 2015). As a result, these methods have gained increasing relevance in both academic research and applied marketing contexts, particularly for examining consumers’ emotional responses.

Despite this methodological progress, a central yet underexamined issue concerns the selection and construction of the stimuli used in neuromarketing studies. Images, words, videos, and brand-related content constitute the primary triggers of the neural and psychophysiological responses analyzed in this field, as they are typically presented as advertisements, products, or logos designed to activate attentional and emotional processes in consumer decision contexts (Plassmann et al., 2012; Venkatraman et al., 2015). Nevertheless, stimulus selection is often treated as a purely technical or procedural matter, receiving limited conceptual, ethical, and normative attention (Poels & Dewitte, 2019; Senior & Lee, 2008).

In this article, the term *stimuli* refers to the experimental materials used in marketing, neuromarketing, and consumer psychology studies—such as images, words, sounds, or other sensory or brand-related content—to elicit affective and cognitive responses in controlled settings (Balconi & Sansone, 2021; Bradley & Lang, 1999, 2007; Lang et al., 2008).

In practice, many neuromarketing studies rely on stimuli developed ad hoc for specific experiments or campaigns, typically validated through emotional-response pretests or pilot testing with convenience samples (Venkatraman et al., 2015). Although this approach offers flexibility, it may also be influenced by researchers’ subjective judgments or by organizations’ strategic objectives, thereby reducing transparency and limiting comparability across studies (Ariely & Berns, 2010; Plassmann et al., 2015; Yang et al., 2018).

While marketing research offers relatively few standardized databases of affective stimuli (e.g., the NeuroBioSense database; Kocaçınar et al., 2024), by contrast, psychology and affective science have developed numerous standardized stimulus databases, typically normed along dimensions such as valence and arousal, which allow affective responses to be induced in a more controlled and comparable manner across studies (Bradley & Lang, 1999; Lang et al., 2008). Despite their widespread use in psychology and neuroscience, these databases remain relatively underutilized in neuromarketing and marketing research (Cenizo, 2022). Their limited adoption is often justified by concerns about ecological validity or by the need to employ brand-specific stimuli (Venkatraman et al., 2015). However, these arguments do not fully address the methodological and ethical implications associated with stimulus selection in a field where clearer responsibility frameworks have been called for to prevent potentially manipulative applications (Ariely & Berns, 2010; Murphy et al., 2008; Stanton et al., 2017).

At the same time, many existing affective databases were originally developed for general affective psychology research rather than for consumption or marketing communication contexts (Lang et al., 2008). Because emotional responses may vary depending on contextual factors—such as brand associations or consumption environments—it is necessary to assess their applicability in marketing research (Aaker et al., 1986; Delatorre et al., 2019; Piqueras-Fiszman & Jaeger, 2014).

Against this background, this study positions stimulus selection as a critical yet underexamined dimension of responsible neuromarketing research. It makes three main contributions. First, it reconceptualizes stimulus selection as a normatively relevant decision with ethical and scientific implications. Second, it critically examines the field’s reliance on ad hoc stimuli and its consequences for transparency, replicability, and participant welfare. Third, it develops a normative framework to guide responsible stimulus selection grounded in principles of ethical transparency, scientific rigor and participant protection.

## 2. Review Approach and Scope

This article adopts a structured narrative review with a mapping component rather than a full systematic review. This approach is appropriate for organizing heterogeneous literature, clarifying conceptual distinctions, and supporting framework development (Grant & Booth, 2009; Snyder, 2019). The aim was not to provide an exhaustive catalogue of all affective datasets, but to identify, classify, and critically assess affective stimulus resources and related datasets potentially relevant to neuromarketing and consumer neuroscience.

The mapping followed four steps. First, KAPODI was used as the baseline source because it provides a systematic and searchable database of emotional stimulus sets published between 1963 and 2020, including resources that are freely available or available upon request and coded according to modality, affective dimensions, rating scales, validation population, country of study, and access conditions (Diconne et al., 2022). Second, because KAPODI is limited to resources available up to 2020, all publications listed in KAPODI were used as seed records for a citation-based update. Citation data were retrieved primarily from Scopus; when Scopus data were unavailable, Google Scholar records were consulted through Publish or Perish 8 (Harzing, 2007). This procedure generated a discovery corpus of 63,952 citing references.

Third, the citation corpus was screened using a semi-automated Python 3.10.4-based procedure designed to detect references to affective databases, identify psychophysiological and neurophysiological techniques, classify broad research domains, and flag papers likely to create, release, validate, adapt, translate, extend, or document affective stimulus resources or related datasets. To reduce false positives, the corpus was restricted to articles and data papers, and then to records classified as potentially creating or documenting a new database, corpus, stimulus set, or dataset. This process yielded 637 candidate references for full-text verification. Fourth, candidate papers were checked against the full text using a predefined coding sheet. Records were retained only when they explicitly created, presented, released, validated, adapted, translated, extended, or documented an affective stimulus resource or related dataset. This process resulted in a final set of 593 retained records.

Because the identified resources differ substantially in function and degree of normativity, they were not treated as equivalent standardized stimulus sets. Instead, they were classified according to their primary research function: normed affective stimulus databases, dynamic or multimodal normed stimulus sets, domain-specific or context-specific stimulus sets, psychophysiological response datasets, and emotion-recognition or affective-computing datasets. This distinction is important because of a normed affective image database, a video-based emotion induction set, a psychophysiological response dataset, and an emotion-recognition benchmark do not offer the same level of direct usability for classical neuromarketing experiments.

For each retained resource, the following information was extracted where available: resource name, publication year, modality, type of resource, affective norms or ratings, rating model or scales, psychophysiological or neural measures, accessibility, potential relevance for neuromarketing, and main limitations. The complete classification dataset generated through the structured narrative mapping is openly available in the Open Science Framework (OSF) repository under the title Bibliographic dataset of standardized stimulus resources (https://doi.org/10.17605/OSF.IO/KMFBS). Supplementary Table S1: Bibliographic dataset of standardized stimulus resources was derived from this full dataset. Affective resources identified through narrative mapping. Citation counts were used only as descriptive indicators of scholarly visibility and not as inclusion criteria, because recently published datasets may be methodologically relevant despite having few citations.

In addition, the review included illustrative estimates of the operational energy use and associated emissions of preliminary instrumental validation phases. These estimates were not intended as life-cycle assessments or precise measurements of neuromarketing experiments, but as sensitivity scenarios to make explicit the assumptions underlying the resource-efficiency argument (Roletto et al., 2026). For fMRI, the Siemens MAGNETOM Trio/Trio Tim 3T system was selected because this scanner family has been documented in published neuromarketing and consumer neuroscience studies (Casado-Aranda et al., 2023; Hsu & Cheng, 2018; Hubert et al., 2018), and its technical specifications report standby and maximum average power values. For EEG, the BioSemi ActiveTwo Mk2 was selected because it has been used in consumer neuroscience research (Boksem & Smidts, 2015; Pozharliev et al., 2015; Telpaz et al., 2015), and manufacturer specifications report the amplifier’s power consumption. Emissions were estimated by multiplying electricity consumption by an illustrative European electricity-emission intensity range of 0.20–0.25 kgCO_2_e/kWh.

## 3. Neuromarketing and the Central Role of Affective Stimuli

The methodological value of neuromarketing depends not only on the sensitivity of the measurement technique, but also on the capacity of the stimulus to evoke the psychological process under study. In consumer neuroscience, stimuli function as the experimental interface between theory and measurement: they translate constructs such as affective appeal, reward expectation, brand attachment, or consumption desire into perceptual events capable of generating measurable neural and physiological responses (Balconi et al., 2014; Karmarkar & Yoon, 2016). For this reason, stimulus selection is not a secondary procedural decision, but a core determinant of construct validity (Senior & Lee, 2008; Somarathna et al., 2024). If the affective and perceptual properties of the stimuli are not adequately specified, controlled, or validated, the resulting responses may reflect uncontrolled features of the material rather than the intended psychological mechanism (Ohme et al., 2009). Consequently, the properties of affective stimuli are central to both experimental design and the interpretation of observed responses.

Research in affective science and emotion research has identified valence and arousal as core dimensions structuring emotional experience (Russell, 1980). These dimensions systematically influence attention, memory, and evaluation, and are widely used to interpret neural and psychophysiological responses. In marketing contexts, positive valence is typically associated with approach tendencies and favorable attitudes, whereas higher arousal has been linked to increased emotional engagement, attentional allocation, and message recall (Bolls et al., 2001; Ohme et al., 2009).

However, many neuromarketing studies rely on broadly defined stimuli—such as “emotional advertisements” or “attractive images”—without specifying their affective properties. This lack of precision limits interpretability and hinders comparability across studies, as observed effects may reflect uncontrolled stimulus characteristics rather than the psychological processes of interest (Poels & Dewitte, 2019). The widespread use of ad hoc stimuli further amplifies this limitation, introducing variability that constrains replicability and cumulative knowledge building (Plassmann et al., 2012).

These limitations highlight the need for more rigorous approaches to stimulus selection, positioning it as a central methodological and conceptual issue in neuromarketing research. This centrality, however, is not uniform across all neuromarketing designs. The methodological importance of stimulus selection depends on the research purpose, the role played by the stimuli in operationalizing the focal construct, and the extent to which their affective, perceptual, or contextual properties may influence the neurophysiological responses under study.

## 4. Standardized Affective Databases in Neuromarketing

The study of emotional responses in neuromarketing and consumer behavior can benefit from the use of previously normed affective stimuli, particularly when the aim is to elicit, compare, or control emotional responses systematically. In this context, standardised affective stimulus databases constitute experimental resources developed across different academic disciplines to provide materials—such as images, words, sounds, facial expressions, or audiovisual sequences—accompanied by normative ratings obtained through systematic procedures of subjective evaluation. These ratings typically describe the expected affective response along dimensions such as valence, arousal, and, in some cases, dominance, in accordance with dimensional models of emotion (Bradley & Lang, 1994; Russell, 1980). Among the most frequently cited resources in the literature is the International Affective Picture System (IAPS; Gerges et al., 2025; Lang et al., 2008), together with other widely used databases, such as the Open Affective Standardized Image Set (OASIS; Kurdi et al., 2017), the Affective Norms for English Words (ANEW; Bradley & Lang, 1999) and its Spanish adaptation (Redondo et al., 2007), the Nencki Affective Picture System (NAPS; Marchewka et al., 2014), and EmoMadrid (Carretié et al., 2019), among others. However, despite their advantages in terms of experimental control and replicability, these databases present important methodological limitations affecting their ecological validity, cultural representativeness, and suitability for constructs specific to consumer behavior (Gerges et al., 2025). The following sections critically examine both dimensions.

### 4.1. Advantages of Standardized Stimuli

The use of standardized affective stimuli offers substantial methodological advantages for research in neuromarketing and consumer behavior. The main advantage lies in their ability to enhance experimental control through the selection of materials that have been previously normed on quantified affective dimensions—primarily valence, arousal, and dominance—based on large participant samples and psychometrically validated (Bradley & Lang, 1994; Lang et al., 2008). This procedure reduces arbitrariness in stimulus selection and allows the observed responses to be attributed more precisely to the manipulated affective content, thereby minimizing the variance explained by the researcher’s subjective decisions (Gerges et al., 2025).

A second advantage lies in their contribution to cross-study comparability and the replicability of findings. The use of widely validated databases allows different laboratories to work with the same stimuli or with materials that are normatively equivalent, thereby facilitating the accumulation of empirical evidence, the systematic comparison of results, and the construction of a robust body of knowledge (Kurdi et al., 2017). This is particularly relevant in areas in which physiological, attentional, or neural responses show substantial interindividual and intercultural variability (Bilucaglia et al., 2024; Gerges et al., 2025). Indeed, the lack of standardization in emotional induction protocols has repeatedly been identified as one of the main obstacles to the comparability of meta-analyses in affective neuroscience (Lindquist et al., 2012).

In addition, these databases make it possible to control physical and perceptual properties of the stimulus that, if left unaddressed, could act as confounding variables in neurophysiological recording. Parameters such as luminance, contrast, visual complexity, the spectral density of low spatial frequencies, lexical frequency, familiarity, and expressive intensity significantly influence brain and autonomic responses independently of affective content (De Cesarei & Codispoti, 2006; Gerges et al., 2025). Such control is especially critical in studies using EEG/ERP, eye-tracking, EDA, or fMRI, where small perceptual differences may contaminate the interpretation of neurophysiological responses and compromise the internal validity of the design (Bilucaglia et al., 2024; Lang et al., 2008).

Finally, from an applied perspective, standardized stimuli provide an efficient starting point for investigating the basic affective processes involved in consumption, such as selective emotional attention, physiological arousal, approach–avoidance motivational systems, and hedonic responding (Casado-Aranda et al., 2023; Cherubino et al., 2019). Although they do not replace contextual validation in real commercial settings, they offer a solid methodological foundation for designing controlled experimental conditions, justifying material selection to academic reviewers, and systematically comparing conditions with different affective loads.

### 4.2. Limitations of Standardized Stimuli

Despite their advantages for experimental control and replicability, standardized affective stimuli present methodological limitations that should be critically considered in the design of neuromarketing and consumer behavior studies. The most widely documented limitation is their restricted ecological validity: because these materials are presented in isolation, out of context, and under highly controlled laboratory conditions, they rarely reproduce the affective complexity of real consumption experiences, in which emotional responses emerge from the interaction of multiple situational factors—such as brand, packaging, price, digital interface, social environment, or decision state (Bilucaglia et al., 2024; Gerges et al., 2025). Dynamic and multimodal stimuli, such as video sequences or virtual reality environments, offer greater ecological realism than static images, although they also introduce additional challenges for standardization (Gerges et al., 2025).

A second limitation concerns the artificiality of the material. In databases of facial and vocal expressions, many expressions are posed, prototypical, or exaggerated, which facilitates their recognition in experimental settings but reduces their similarity to the spontaneous affective displays observed in real consumption situations (Tottenham et al., 2009). Similarly, isolated images or words may induce high-intensity basic affective states, but they are less likely to evoke emotions that are functionally relevant to consumption—such as curiosity, interest, trust, boredom, frustration, or desire—which tend to exhibit subtler activation profiles within the valence–arousal dimensional space (Kurdi et al., 2017; Russell, 1980).

In addition, most normative databases are affected by demographic, cultural, and temporal biases. Original validation studies are often based on young, university-educated, Western samples—commonly referred to in the English-language literature as WEIRD samples (Western, Educated, Industrialized, Rich, Democratic)—which limits the generalizability of affective norms to other age groups, cultures, or market segments (Gerges et al., 2025). Moreover, some stimuli may have lost cultural relevance, particularly in visual databases developed decades ago, thereby affecting their evocative capacity in contemporary participants (Gerges et al., 2025; Lang et al., 2008).

Finally, from an applied marketing perspective, a key limitation is that traditional normative dimensions—valence, arousal, and dominance—do not always map onto core consumer behavior constructs, such as purchase intention, brand attitude, perceived value, engagement, or loyalty (Casado-Aranda et al., 2023; Cherubino et al., 2019). Thus, although standardized stimuli are useful for isolating basic affective processes under controlled conditions, their use in neuromarketing often requires sample-specific pretesting and, where appropriate, adaptation to realistic commercial settings to support the external validity of the findings. Table 1 summarizes these strengths and limitations, particularly in relation to experimental control, replicability, ecological validity, representativeness, and fit with marketing-specific constructs.

## 5. The Ad Hoc Stimulus Selection in Neuromarketing

Despite the consolidation of affective databases as a methodological benchmark in emotion research, neuromarketing studies have predominantly relied on ad hoc stimuli—designed or selected to represent the specific product, brand, advertisement, or consumer experience under investigation (Plassmann et al., 2012; Venkatraman et al., 2015). This choice reflects not merely operational convenience, but a fundamental requirement to align experimental materials with the applied research questions that define consumer neuroscience (Smidts et al., 2014). However, the contextual flexibility that makes ad hoc stimuli ecologically relevant also introduces methodological trade-offs in experimental control, stimulus validation, and cross-study comparability—a tension that demands critical appraisal before assuming either their ecological superiority or their scientific adequacy (Ariely & Berns, 2010).

### 5.1. Advantages of Ad Hoc Stimuli

Ad hoc-designed stimuli offer a key advantage over standardised stimuli: they allow researchers to maximise the correspondence between the experimental material and the real-world consumption phenomenon under investigation. In neuromarketing, many affective responses do not arise from generic emotional stimuli, but rather from specific configurations of brand, product, price, packaging, interface, advertisement, or purchase experience (Alsharif et al., 2021; Wang et al., 2024). For this reason, ad hoc stimuli enable a more precise operationalisation of applied constructs such as brand attitude, purchase intention, engagement, trust, desire, perceived value, and ease of navigation (Esch et al., 2012; Plassmann et al., 2012).

A second advantage lies in their greater ecological validity. By incorporating elements inherent to the market context—such as visual identity, advertising claims, product design, digital environment, music, narrative, or interface interaction—these stimuli can more faithfully reproduce the conditions under which consumer responses are formed (Ariely & Berns, 2010; Lim, 2018). This is particularly relevant in studies using eye-tracking, EEG, EDA, or fMRI, where the interpretation of neurophysiological responses depends on whether the stimulus activates psychological processes that closely resemble those occurring in real commercial settings (Hakim & Levy, 2018; Khushaba et al., 2013).

In addition, ad hoc stimuli make it possible to tailor the experimental design to the target population, the sector under investigation, and the specific hypothesis being tested. This flexibility is especially valuable in applied academic and professional research, where standardised stimuli may be too generic, culturally distant, or insufficiently sensitive to emotions of commercial relevance, such as curiosity, boredom, frustration, aesthetic attraction, credibility, or brand identification (Alsharif et al., 2021; Plassmann et al., 2012). In this sense, ad hoc stimuli not only enhance the applied relevance of the study, but may also strengthen construct validity when they are carefully pretested and validated against the key dimensions of the targeted construct (Ariely & Berns, 2010; Venkatraman et al., 2015).

### 5.2. Limitations of Ad Hoc Stimuli

Despite their greater contextual relevance, ad hoc stimuli present important methodological limitations. The main one is the potential loss of experimental control. By incorporating multiple realistic elements—such as brand, colour, text, product, price, visual design, music, or narrative—the risk increases that the observed responses may be driven by uncontrolled variables rather than by the psychological mechanism under investigation (Ariely & Berns, 2010; Lim, 2018). For example, greater physiological arousal in response to an advertisement may be attributable to its emotional content, but also to brand familiarity, visual complexity, prior preference, or the novelty of the stimulus (Esch et al., 2012; Hakim & Levy, 2018).

A primary concern is the arbitrariness inherent in stimulus construction. Stimuli are often defined based on subjective judgments, organizational priorities, or contextual constraints rather than on explicit theoretical frameworks of emotion. This weakens the transparency of the relationship between stimulus properties and observed neural or physiological responses, making it difficult to determine which affective dimensions are being activated and the underlying mechanisms driving such activation (Karmarkar & Plassmann, 2019). As a result, findings tend to be context-dependent and limited in external validity (Kocaçınar et al., 2024), highlighting the need for greater standardization to allow for hypothesis validation (Balconi & Sansone, 2021; Cenizo, 2022).

The reliance on ad hoc stimuli also poses significant challenges for replicability and cumulative knowledge development. When stimuli are unique, insufficiently documented, or inaccessible, replication becomes difficult, restricting systematic comparison across studies and slowing theoretical consolidation (Wang et al., 2024; Yang et al., 2018). These challenges are particularly acute in applied contexts, where copyright and confidentiality constraints further limit transparency and independent verification (Stanton et al., 2017).

Likewise, ad hoc stimuli require a rigorous process of pretesting and validation. It is not sufficient for the material to be realistic or appealing; it must be shown to manipulate the construct of interest effectively and not to introduce unwanted differences in variables such as familiarity, prior liking, credibility, comprehension, visual complexity, personal relevance, or baseline purchase intention (Venkatraman et al., 2015). Without such prior validation, any gain in realism may come at the cost of reduced inferential precision, because observed effects can no longer be confidently attributed to the construct of interest rather than to uncontrolled differences between stimuli (Ariely & Berns, 2010).

As a consequence of these pretesting requirements, the development of ad hoc stimuli increases experimental burden and resource consumption, as these techniques often require substantial funding and extensive timelines (Plassmann et al., 2012). Pretesting procedures require additional samples, time, and financial resources, often duplicating efforts already undertaken in prior research (Cenizo, 2022). From a sustainability perspective, this redundancy raises concerns regarding the efficient use of scientific and human resources, highlighting the need for shared materials and open science practices (Christensen et al., 2022; EFPA, 2025).

Ad hoc stimulus design also entails ethical implications. The deliberate construction of stimuli aimed at maximizing specific emotional responses—such as heightened attention or physiological arousal—may unintentionally exceed acceptable thresholds of influence, particularly when such processes operate at a non-conscious level (Ariely & Berns, 2010; Christensen et al., 2022; Ferrell et al., 2025). The incorporation of stimuli delivered through VR, AR, or metaverse environments further heightens this concern, as these technologies may increase the sense of presence—the subjective illusion of “being there” in the digital environment (Madary & Metzinger, 2016)—and perceptual realism, whereby mediated stimuli are perceived as real and elicit genuine emotional responses in accordance with the “law of apparent reality” (Mhaidli et al., 2023; Poels & Dewitte, 2019). In addition, these technologies facilitate experiences of embodiment, in which users identify a virtual body as their own, potentially altering behaviour and self-perception through the Proteus effect (Madary & Metzinger, 2016; Yee et al., 2009). Finally, psychological proximity to the stimulus is intensified, since in these immersive environments events are not passively observed on a screen, but rather experienced as if they were happening directly to the user, which may result in deeper psychological and physiological impacts than those associated with traditional media (Mhaidli et al., 2023). This concern is especially salient for vulnerable populations, who may be less able to critically evaluate persuasive content (Bulley et al., 2018; Javor et al., 2013).

Moreover, unlike standardized stimuli, ad hoc materials lack a documented history of use, which limits the ability to anticipate potential adverse effects and may conflict with principles of harm minimization and participant protection (Lang et al., 2008; Senior & Lee, 2008).

Taken together, these limitations suggest that the prevalence of ad hoc stimuli is not merely a methodological choice but a broader normative issue with implications for interpretability, replicability, and research ethics. Table 1 summarizes this trade-off, showing how the ecological validity, applied relevance, and contextual flexibility of ad hoc stimuli must be balanced against limitations in experimental control, replicability, validation resources, and ethical oversight.

## 6. Affective Resources for Neuromarketing

Considering these limitations, affective stimulus databases and related validated resources represent a theoretically grounded but still underexploited methodological asset for neuromarketing. These resources should not be understood as a homogeneous category. Some provide normed affective stimuli with ratings of valence, arousal, dominance, or discrete emotions; others consist of dynamic or multimodal materials, psychophysiological response datasets, eye-tracking datasets, or emotion-recognition benchmarks. This distinction is important because these resources differ in their degree of normativity, direct experimental usability, ecological validity, and relevance for classical neuromarketing designs (Diconne et al., 2022; Gerges et al., 2025). Table 2 therefore presents a representative classification of selected affective resources, while the broader set of resources identified through the narrative mapping is reported in Supplementary Table S1.

A central advantage of normed affective stimulus databases lies in their capacity to enhance experimental control and facilitate comparability across studies. Empirically validated affective ratings, commonly obtained through instruments such as the Self-Assessment Manikin (SAM), allow researchers to select stimuli according to predefined emotional dimensions and to interpret neurophysiological or behavioral responses with greater precision (Bradley & Lang, 1994; Lang et al., 2008). However, this advantage applies most directly to resources specifically designed as normed stimulus databases; psychophysiological datasets or emotion-recognition benchmarks may be more useful for modelling, benchmarking, or secondary analysis than for direct reuse as stimuli in classical neuromarketing experiments.

In addition, affective resources can support transparency and replicability by enabling stimulus traceability, reuse, and clearer reporting of stimulus properties, in line with broader open-science principles regarding the documentation and reuse of research materials (Nosek et al., 2015; Wilkinson et al., 2016). In the specific case of affective stimuli, searchable and normed databases can facilitate comparison across studies and reduce the need for extensive preliminary affective validation when the research question concerns general affective or cognitive mechanisms (Diconne et al., 2022; Lang et al., 2008). However, in applied marketing contexts, additional pretesting may still be necessary to assess brand associations, product relevance, cultural interpretation, or contextual fit, because affective responses can vary across populations, contexts, and stimulus meanings (Branco et al., 2023; Delatorre et al., 2019; Gerger et al., 2014).

Despite these benefits, their adoption in neuromarketing appears to remain limited in many applied contexts, particularly when researchers prioritize brand-specific or commercially realistic stimuli over normed affective materials (Cenizo, 2022; Karmarkar & Plassmann, 2019). Concerns about ecological validity—especially the absence of direct links to brands, products, or commercial environments—are often cited as a reason for relying on ad hoc stimuli. Yet this concern should not lead to a simple opposition between standardized and ad hoc materials. Standardized resources are especially useful when the aim is to isolate general affective mechanisms, whereas context-specific stimuli may be preferable when the research question depends on brand meaning, product category, cultural symbolism, or consumer context (Karmarkar & Plassmann, 2019).

Accordingly, standardized affective databases and related validated resources should be understood as complementary tools within a staged research strategy. Normed databases may be particularly valuable in exploratory or mechanism-focused phases, while context-specific or ad hoc stimuli may be introduced in later stages to test whether the identified mechanisms generalize to realistic commercial settings (Plassmann et al., 2012).

Beyond methodological considerations, standardized databases also contribute to research efficiency and sustainability by reducing redundant stimulus development, minimizing participant burden, and streamlining data collection processes (Ferrell et al., 2025). Furthermore, their documented properties facilitate ethical evaluation by enabling more accurate anticipation of participant responses (Ferrell et al., 2025; NMSBA, 2013).

Nevertheless, their application in marketing contexts requires careful consideration. Affective responses may vary depending on factors such as cultural background, product category, and brand associations, highlighting the need for empirical validation (Branco et al., 2023; Delatorre et al., 2019; Gerger et al., 2014). This also points to an important avenue for future research: the development of affective databases specifically tailored to marketing applications, combining standardization with contextual relevance.

Overall, standardized affective databases offer substantial potential to enhance methodological rigor, transparency, and sustainability in neuromarketing research, yet remain underutilized within the field.

## 7. Ethical, Scientific Responsibility, and Sustainability Implications of Stimulus Selection

Stimulus selection in neuromarketing can be understood as a normatively relevant consideration that extends beyond purely methodological concerns. The distinction between ad hoc and standardized affective stimuli appears to carry implications for research ethics, scientific responsibility, and the sustainability of research practices. In addition, even when standardized databases are employed, their contextual validity within marketing environments requires careful consideration. These aspects suggest that stimulus selection plays a meaningful role in shaping more responsible neuromarketing research practices.

Collectively, these considerations inform the development of the normative framework presented in the following section.

### 7.1. Ethical Transparency and the Regulation of Emotional Influence

One relevant ethical challenge in neuromarketing lies in its capacity to access non-conscious emotional and cognitive processes. Unlike self-report methods, neuromarketing techniques capture affective responses that participants may not be able to identify or articulate, thereby increasing the risk of opaque emotional influence (Ariely & Berns, 2010). Within this context, stimulus selection becomes a critical site of ethical responsibility.

Ad hoc stimulus design may amplify this risk. When stimuli are intentionally constructed to maximize specific affective responses—such as heightened arousal or attentional capture—key design decisions remain difficult to observe and evaluate externally. In the absence of systematic affective characterization, it becomes challenging to determine which emotions are being elicited, at what intensity, and with what potential unintended effects (Ferrell et al., 2025; Stanton et al., 2017).

In contrast, standardized affective stimuli enhance ethical transparency. The availability of normative ratings of valence and arousal allows researchers to explicitly justify stimulus selection and facilitates ethical oversight by review boards and regulatory bodies (Ferrell et al., 2025; NMSBA, 2013). In this sense, standardization operates as a mechanism for constraining unjustified or excessive emotional influence, aligning experimental design with principles of accountability and transparency.

### 7.2. Scientific Responsibility, Replicability, and Epistemic Robustness

Scientific responsibility is particularly salient in emerging fields such as neuromarketing, where the credibility of the field depends on the robustness and reproducibility of its findings. The widespread reliance on ad hoc stimuli poses a structural challenge to these objectives (Plassmann et al., 2015).

When stimuli are unique, insufficiently documented, or inaccessible, empirical findings become tightly coupled to specific experimental contexts. This limits the possibility of replication, constrains cross-study comparability, and hinders the accumulation of coherent theoretical knowledge. From an epistemological perspective, stimulus selection thus directly affects the robustness and generalizability of the knowledge produced (Balconi & Sansone, 2021; Plassmann et al., 2015).

Standardized affective databases offer a pathway toward greater scientific responsibility by enabling direct replication of stimuli and facilitating systematic comparison across studies (Lang et al., 2008). Their use aligns with open science practices, including transparent reporting and the reuse of validated materials, thereby supporting cumulative knowledge development (EFPA, 2025).

However, replicability depends not only on the availability of standardized stimuli but also on the stability of their affective properties across contexts. As previously noted, normative ratings derived from general populations may not fully generalize to specific marketing environments, underscoring the need for contextual validation alongside standardization.

### 7.3. Sustainability and Resource Efficiency in Experimental Research

Sustainability represents an increasingly relevant dimension in the evaluation of research practices, not only because of their environmental impact, but also because of their implications for the efficient use of economic, human, technical, and temporal resources (Ioannidis et al., 2014). In neuromarketing, this issue becomes particularly relevant when the development of ad hoc stimuli requires additional stages of selection, piloting, calibration, or instrumental validation, especially in studies relying on resource-intensive techniques such as fMRI, EEG, eye-tracking, or other psychophysiological measures (Ariely & Berns, 2010; Plassmann et al., 2012). These stages may increase the use of participants, laboratory time, and experimental infrastructure, although such costs are rarely made explicit in methodological reports.

From this perspective, the argument is not that every ad hoc stimulus necessarily increases the environmental footprint of a study, but rather that, when its development requires additional validation sessions using instrumental techniques, these stages generate additional energy consumption that should be transparently reported. This reasoning is consistent with broader calls to reduce research waste, reuse existing resources when scientifically appropriate, and make the environmental costs of neuroscience research more visible (Ioannidis et al., 2014; Roletto et al., 2026). To illustrate this issue, Table 3 presents indicative scenarios of the operational energy consumption associated with preliminary stages of instrumental validation in neuromarketing. These estimates are not intended to constitute precise empirical measurements or full life-cycle assessments, but rather sensitivity scenarios aimed at making transparent the order of magnitude of the consumption potentially associated with these practices.

Energy consumption was estimated using the following general expression:

Energy consumption (kWh) = Σ (component power in kW × hours of use)

The associated operational emissions were estimated by applying an illustrative range of electricity emissions intensity:

Emissions (kgCO_2_e) = energy consumption (kWh) × emission factor (kgCO_2_e/kWh)

In experimental research using fMRI, studies may include different forms of preliminary paradigm validation—for example, pilot studies, preliminary functional tests, or functional localizers—before data collection for the main experiment. These stages make it possible to assess whether the stimuli elicit the expected responses, whether the experimental design is technically feasible within the scanner, and whether the acquisition parameters are appropriate for detecting the BOLD signal of interest (Poldrack et al., 2008). In more sophisticated approaches, stimulus selection may even be conducted using real-time fMRI, employing the BOLD response in predefined regions of interest to optimize stimuli before the main protocol (Leeds & Tarr, 2016).

From a methodological design perspective, fMRI pilot stages typically involve small, exploratory samples aimed at assessing paradigm feasibility rather than estimating definitive inferential effects (Dougherty et al., 2015; Mumford & Nichols, 2008). Considering functional acquisition sessions of approximately 40–60 min, together with additional time for preparation, positioning, and recovery, an approximate total duration of 90 min per participant constitutes a reasonable operational assumption for constructing an illustrative consumption scenario.

To avoid a generic estimate of fMRI energy consumption, a scanner documented in neuromarketing and consumer neuroscience studies was adopted as a reference: the 3-Tesla Siemens MAGNETOM Trio/Trio Tim. This scanner family has been used in research on audiovisual advertising, word-of-mouth communication following product crises, and online trust (Casado-Aranda et al., 2023; Hsu & Cheng, 2018; Hubert et al., 2018). The technical datasheet for the Siemens Trio A Tim System specifies a standby power of 13 kVA and a maximum average power of 54 kVA (Siemens Medical Solutions, 2026). Because these values are expressed in kVA rather than kW, the estimates are presented as approximations based on apparent power; under the conservative assumption of a power factor equal to 1, these values are treated as kW-equivalents for illustrative purposes only.

Applying these parameters to an fMRI pilot stage consisting of 12 participants and 90 min sessions, the cumulative energy consumption would range from 234 kWh, if only the system’s standby power is considered, to 972 kWh, if the maximum average power reported in the technical datasheet is applied. Using an illustrative range of electricity emissions intensity of 0.20–0.25 kgCO_2_e/kWh, this preliminary stage would correspond approximately to 46.8–58.5 kgCO_2_e in the low scenario and to 194.4–243.0 kgCO_2_e in the high scenario. These figures should not be interpreted as empirical measurements of the actual energy consumption of an fMRI session, but rather as sensitivity scenarios that help make visible the potential operational consumption associated with preliminary stages of instrumental validation.

Analogously, the use of EEG substantially reduces the energy intensity per session, but does not entirely eliminate the resources associated with preliminary stimulus validation. In marketing, Kristofferson and Dunn (2023) explicitly employ a preliminary EEG study within a research program on brand displacement, showing that EEG can be used as a preliminary stage in consumer behavior studies. However, this reference should be used to justify the methodological relevance of the procedure, not to estimate energy consumption, since it does not provide a detailed temporal structure of the EEG recording.

To estimate the minimum instrumental cost of a preliminary EEG stage, Schettino et al. (2020) provide a more precise basis. These authors conducted two EEG pilot studies before the main experiment; in Pilot 1, 16 participants took part, and the task included 576 trials distributed across eight blocks of approximately 6 min and 20 s, equivalent to around 50.7 min of effective EEG recording per participant. Recording was performed using a BioSemi ActiveTwo system, 64 Ag/AgCl electrodes, and a sampling rate of 256 Hz, confirming that this was a pilot stage using full EEG equipment, rather than a merely behavioral pretest.

Given that 16 participants took part and each completed approximately 50.7 min of effective recording, a pilot stage of this kind would involve 13.52 cumulative hours of instrumental use. If this experimental load is applied to the BioSemi ActiveTwo/Active-Two, which has been used in studies published in the *Journal of Marketing Research* (Boksem & Smidts, 2015; Pozharliev et al., 2015; Telpaz et al., 2015), a minimum estimate of the amplifier’s electricity consumption can be obtained. According to the manufacturer’s technical specifications, the BioSemi ActiveTwo Mk2 has a consumption of 4 W at 280 channels. Using this value as a conservative upper bound for the EEG amplifier, the consumption associated exclusively with the effective recording time would be 54.08 Wh, that is, 0.054 kWh. Applying the same illustrative range of electricity intensity, the operational emissions attributable to the EEG amplifier would be 0.0108–0.0135 kgCO_2_e, equivalent to 10.8–13.5 gCO_2_e.

This estimate should be interpreted as the minimum impact associated exclusively with the EEG amplifier during the effective recording time, not as the total impact of an EEG session. The calculation excludes the acquisition computer, monitors, lighting, laboratory climate control, participant preparation, cleaning of materials, consumables, auxiliary infrastructure, and possible repetitions or discarded sessions. Its usefulness in a critical narrative review therefore lies not in magnifying the impact of the EEG amplifier in isolation, but in showing the need to transparently report the assumptions, boundaries, and resources involved in preliminary stages of experimental validation.

Taken together, these scenarios suggest that preliminary stages of instrumental validation may generate additional energy costs, especially when they involve high-intensity neuroimaging techniques such as fMRI. The central issue is not that the use of ad hoc stimuli is inherently unsustainable, but rather that their associated costs should be made explicit when they require additional piloting, calibration, or instrumental sessions.

### 7.4. Participant Welfare and Harm Minimization

Participant welfare can be understood as a fundamental principle in research involving human subjects. In neuromarketing, this principle is particularly salient due to the use of emotionally evocative stimuli and the measurement of non-conscious responses (Ferrell et al., 2025).

Repeated exposure to intense or insufficiently characterized stimuli may induce discomfort, stress, or unintended adverse reactions. In immersive environments, however, the ethical evaluation of participant welfare requires a more differentiated analysis. Not all risks associated with virtual reality (VR), augmented reality (AR), or metaverse-based studies arise directly from stimulus selection itself. Some risks are related to the affective, perceptual, or semantic properties of the stimulus, whereas others derive from the exposure medium, the device used, the interface design, the data collected, or the broader experimental protocol (Madary & Metzinger, 2016).

First, stimulus-related risks concern the content and properties of the material presented to participants. These include emotional intensity, negative valence, threat-related content, persuasive cues, product-crisis scenarios, aversive imagery, or culturally sensitive representations. In these cases, responsible stimulus selection requires documenting affective properties, pretesting emotional intensity, minimizing unnecessary distress, and ensuring that the stimulus is appropriate for the research question. Standardized affective stimuli provide a clear advantage in this regard, as their documented properties and prior use allow researchers to better anticipate participant responses and implement appropriate safeguards (Branco et al., 2023).

Second, medium- and device-related risks are not necessarily caused by the stimulus content, but by the immersive technology through which the stimulus is delivered. The literature has documented adverse physiological effects associated with immersive exposure, including cybersickness, nausea, disorientation, visual fatigue, and oculomotor discomfort (Conner et al., 2022; Simón-Vicente et al., 2024). From a psychological perspective, immersive environments may also intensify affective responses, induce experiences of embodiment, temporarily alter self-perception, and, in some cases, be associated with symptoms of depersonalization or derealization (Barreda-Ángeles & Hartmann, 2023; Madary & Metzinger, 2016; Yee et al., 2009). These risks should therefore be addressed through device calibration, exposure-time limits, safety instructions, exclusion criteria, distress monitoring, and the possibility of immediate withdrawal.

Third, protocol- and data-related risks concern the broader experimental environment. In consumer behaviour and marketing research, immersive technologies may involve hyper- personalization, emotionally intensive advertising, persuasive interaction design, and the collection of behavioural, biometric, gaze-tracking, motion, voice, or contextual data (Dwivedi et al., 2023; Harborth & Pape, 2021; Mhaidli et al., 2023). These risks are not reducible to stimulus selection alone and require enhanced informed consent procedures, clear data-governance rules, secure storage, minimization of unnecessary data collection, debriefing, and robust data protection measures (Christopoulos et al., 2021; Ferrell et al., 2025).

Returning specifically to stimulus selection, ad hoc stimuli often lack prior documentation, increasing uncertainty regarding their potential effects—especially in vulnerable populations or culturally diverse contexts. From a normative standpoint, prioritizing stimuli with validated affective properties aligns with the principle of harm minimization and with the obligation to protect participants, particularly when dealing with populations that may be more susceptible to emotional influence (Ferrell et al., 2025). In immersive environments, this requirement becomes even more important, because stimulus-related risks may interact with medium-, device-, protocol-, and data-related risks.

### 7.5. Synthesis: Stimulus Selection as a Normatively Consequential Decision

Taken together, these arguments suggest that stimulus selection in neuromarketing is not a neutral or purely technical decision. It is a normatively consequential choice that directly affects ethical transparency, scientific robustness, resource efficiency, and participant welfare.

Treating stimulus selection as a secondary methodological issue obscures its impact on the research process and limits the capacity of neuromarketing to contribute to responsible marketing practices. By contrast, recognizing its normative significance repositions stimulus selection as a key point of intervention for enhancing ethical, transparent, and sustainable research.

Building on this premise, the following section develops a normative framework for responsible stimulus selection, integrating the ethical, scientific, and practical considerations outlined above.

## 8. Toward a Normative Framework for Responsible Stimulus Selection in Neuromarketing

Building on the considerations outlined in Section 7, this section develops a normative framework to guide stimulus selection in neuromarketing from a responsibility-oriented perspective. The framework is grounded in the premise that stimulus selection is not a neutral technical decision but a methodological choice with ethical, epistemic, and sustainability implications.

However, the relevance of stimulus selection should not be understood as uniform across all neuromarketing studies. Its importance depends on the purpose of the research, the role that stimuli play in operationalizing the main construct, and the extent to which their affective, perceptual, or contextual properties may influence the neurophysiological responses observed. This distinction is consistent with consumer neuroscience frameworks that emphasize that neurophysiological methods are most informative when they are aligned with the theoretical construct and research question under investigation (Karmarkar & Plassmann, 2019; Plassmann et al., 2012). Thus, stimulus selection is particularly critical when stimuli constitute the central manipulation of the study—for example, in research on emotion, trust, advertising congruence, threat, reward, or brand responses—whereas it may play a more instrumental role in studies focused primarily on technical validation, signal processing, method comparison, or the analysis of individual differences.

The contribution of the framework does not lie in proposing entirely new ethical principles, but rather in translating and articulating principles already present in research ethics, open science, reproducibility, and sustainability into a specific methodological issue that usually receives less systematic attention: the selection, validation, documentation, and reporting of stimuli in neuromarketing. Unlike general frameworks on neuromarketing ethics or responsible research conduct, the present framework focuses on how to decide between standardized and ad hoc stimuli, what minimum information should be documented, and under what conditions stimulus customization may be considered methodologically justified.

Accordingly, four interrelated normative principles are proposed, each accompanied by operational guidelines. Together, they aim to provide a structured basis for aligning neuromarketing practices with standards of transparency, scientific robustness, and responsible research conduct across both academic and applied contexts. The novelty of the framework does not lie in proposing entirely new ethical principles, but rather in integrating principles derived from responsible research and innovation, open science, reproducibility, transparency in the reporting of materials, and the reduction in research waste into a framework specifically designed for the selection, documentation, and reporting of stimuli in neuromarketing (Ioannidis et al., 2014; Munafò et al., 2017; Nosek et al., 2015; Wilkinson et al., 2016). Unlike these broader frameworks, which are oriented toward the responsible governance of research, the openness of data and materials, or the improvement of reproducibility, the proposed framework focuses specifically on how to decide between standardized and ad hoc stimuli, what minimum information should be documented, and under what conditions stimulus customization may be considered methodologically justified.

### 8.1. Principle 1: Ethical Transparency in Stimulus Selection

Principle

Stimulus selection should be transparent, theoretically grounded, and explicitly justifiable in terms of the affective responses it is intended to elicit.

Justification

Neuromarketing methods enable access to non-conscious affective and cognitive processes, which raises persistent concerns about autonomy, manipulation, and the opacity of commercial influence (Ariely & Berns, 2010; Murphy et al., 2008). In this context, insufficiently documented stimulus selection limits ethical scrutiny and may obscure the mechanisms through which responses are elicited, making transparency in affective characterization a necessary condition for ethical accountability in neuromarketing research (Murphy et al., 2008; Stanton et al., 2017).

This requirement can be observed in neuromarketing studies on online trust. For example, Hubert et al. (2018) used online offer configurations designed to elicit trust evaluations in digital contexts. In this type of design, stimuli do not function as neutral materials, but rather as experimental configurations that incorporate specific cues of trust or distrust. Therefore, ethical transparency requires documenting which elements of the stimulus operate as persuasive cues, how they were experimentally manipulated, and whether their capacity to elicit the expected responses was assessed beforehand (Hubert et al., 2018).

Operational guidelines

- Prioritize the use of standardized affective stimuli with normatively established emotional properties.
- Explicitly report relevant affective dimensions (e.g., valence, arousal) associated with the stimuli.
- When ad hoc stimuli are employed, provide a clear theoretical justification and systematic affective characterization.

### 8.2. Principle 2: Scientific Responsibility and Replicability

Principle

Stimulus selection should support replicability, cross-study comparability, and the cumulative development of knowledge in neuromarketing.

Justification

The use of idiosyncratic or poorly documented stimuli constrains replication and limits the integration of findings across studies, thereby weakening the epistemic robustness of the field (Balconi & Sansone, 2021; Plassmann et al., 2015). In emerging domains such as neuromarketing, these limitations directly affect scientific credibility and theoretical consolidation.

The relevance of this issue is evident in audiovisual advertising studies such as that by Casado-Aranda et al. (2018), in which neural effects depend on specific combinations of gender-targeted products and congruent or incongruent voices. In this case, replicability depends not only on describing the fMRI task or acquisition parameters, but also on documenting the product–voice combinations, the criteria used to define congruence or incongruence, the duration of the stimuli, and their perceptual properties. Without this information, comparisons across studies remain limited even when the neurophysiological protocol is adequately reported.

Operational guidelines

- Use standardized stimuli when investigating general affective or cognitive mechanisms.
- Ensure accessibility or detailed documentation of stimuli to enable replication.
- Treat the development of ad hoc stimuli as a theoretically justified methodological choice rather than a default practice.

### 8.3. Principle 3: Sustainability and Resource Efficiency

Principle

Stimulus selection should promote efficient and sustainable use of research resources, minimizing redundant experimental procedures.

Justification

As discussed in Section 7.3, the repeated development and validation of ad hoc stimuli entails cumulative consumption of economic, human, and energy resources (Ariely & Berns, 2010; Ferrell et al., 2025). In the context of increasing expectations regarding responsible research practices, such redundancy warrants critical evaluation when validated alternatives are available (Stanton et al., 2017). Under this premise, the validity of a study depends not only on its technical rigor, but also on its capacity to generate reusable knowledge (Wilkinson et al., 2016) that, for example, justifies the investment of resources in complex and costly infrastructures such as functional magnetic resonance imaging (fMRI).

Sustainability, therefore, does not imply rejecting ad hoc stimuli, but rather justifying their use when they provide ecological, applied, or contextual validity that standardized materials cannot offer (Ioannidis et al., 2014). In studies such as that by Hsu and Cheng (2018), where the phenomenon under investigation depends on specific information about product crises and word-of-mouth (WOM) communication, ad hoc stimuli may be methodologically justified. However, precisely to avoid research waste, their development should be accompanied by an explicit description of the selection and validation procedures in accordance with international transparency standards (Nosek et al., 2015). An example of this rigor can be observed in the use of instrumental pilot sessions, such as fMRI pilot testing, to ensure that the materials effectively elicit the expected neural activation before the main study begins, thereby mitigating the risk of obtaining spurious results due to flawed designs (Hsu & Cheng, 2018; Munafò et al., 2017).

This consideration is especially relevant in studies that use negative stimuli, product-crisis scenarios, threat messages, aversive content, or images of high emotional intensity. In such cases, stimulus selection should incorporate explicit criteria for controlling affective intensity, minimizing harm, enabling withdrawal, providing debriefing, and excluding materials that are unnecessarily disturbing. Prior documentation of the affective properties of stimuli—for example, valence, arousal, dominance, or emotional intensity—makes it possible to anticipate more accurately the type and degree of emotional response expected, particularly when negative or highly activating materials are used (Bradley & Lang, 1994; Kurdi et al., 2017). Likewise, this control of intensity seeks to reduce analytical flexibility, ensuring that the results are not artifacts derived from uncontrolled extreme emotional stimulation (Munafò et al., 2017). This information facilitates the ethical evaluation of the study, as research frameworks involving human participants require foreseeable risks to be identified, minimized, and documented before experimental exposure (Emanuel et al., 2000; World Medical Association, 2025).

Operational guidelines

- Reuse validated affective stimuli to reduce the need for additional pretesting.
- Consider the cumulative resource and energy implications of experimental design decisions.
- Incorporate sustainability criteria into methodological planning in neuromarketing research.

### 8.4. Principle 4: Protection of Participant Welfare

Principle

Stimulus selection should prioritize participant welfare and minimize exposure to unnecessary emotional risk.

Justification

Exposure to emotionally intense or insufficiently characterized stimuli may produce unintended adverse effects (Christensen et al., 2022). This concern is particularly relevant in neuromarketing, where non-conscious responses are measured and where participants may not fully anticipate their reactions (Ferrell et al., 2025).

Operational guidelines

- Prioritize stimuli with documented affective properties and prior validated use.
- Avoid unnecessary exposure to highly intense stimuli unless required by the research objectives.
- Integrate participant welfare considerations into ethical review and study design.

### 8.5. Operationalizing the Framework: Checklist and Decision-Making Scheme

To prevent the proposed framework from remaining at a merely declarative level, the four principles outlined above are translated into an operational list of minimum criteria for the selection, documentation, and justification of stimuli in neuromarketing studies. This operationalization makes it possible to distinguish between two methodological situations: (a) studies aimed at examining general affective or cognitive mechanisms, in which standardized stimuli should constitute the preferred option; and (b) studies aimed at brand-, product-, platform-, message-, or consumption-context-specific phenomena, in which ad hoc stimuli may be fully justified, provided that their properties, validation pro-cedure, and usage restrictions are documented.

**Figure 1:**
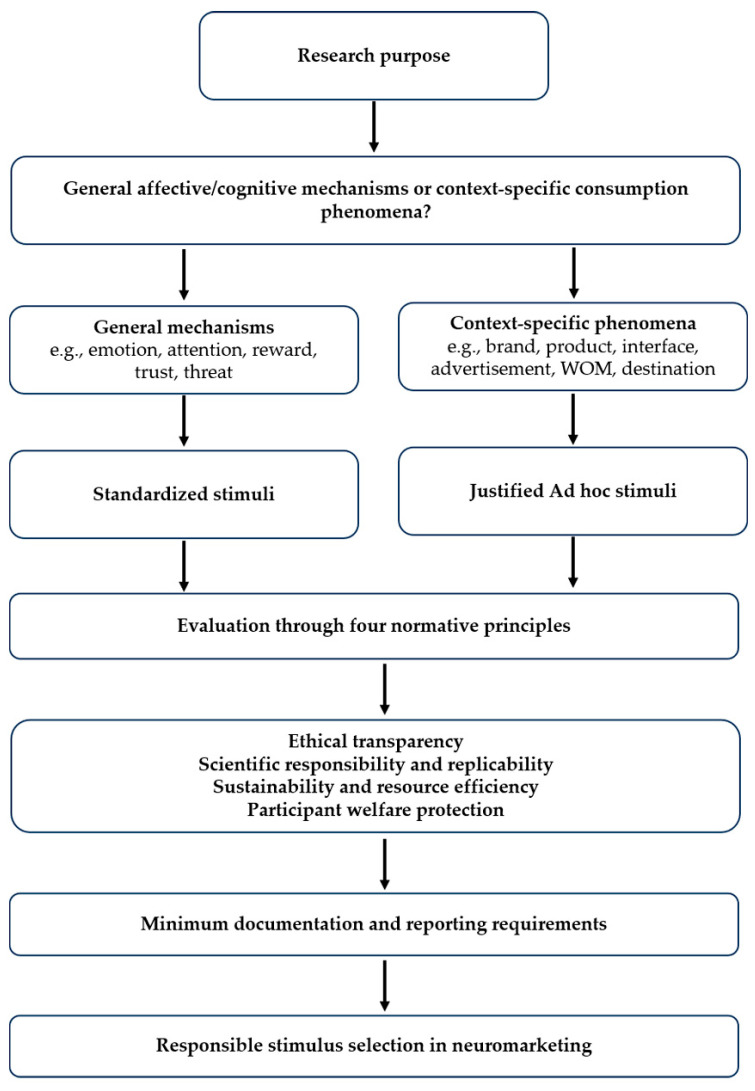
Normative-operational framework for responsible stimulus selection in neuromarketing. Note: The framework starts from the research purpose, distinguishes between standardized and ad hoc stimuli, evaluates both options through four normative principles, and concludes with minimum documentation and reporting requirements.

Figure 1 summarizes the proposed normative-operational framework for responsible stimulus selection in neuromarketing. The framework starts from the research purpose, distinguishes between studies focused on general affective or cognitive mechanisms and those focused on context-specific consumption phenomena, and links this distinction to the choice between standardized and ad hoc stimuli. In both cases, stimulus selection should be evaluated through the four normative principles and documented according to minimum reporting requirements.

As shown in Figure 1, the framework does not prescribe standardized stimuli in all cases. Rather, it proposes a conditional decision logic: standardized stimuli are preferable when the aim is to examine general affective or cognitive mechanisms, whereas ad hoc stimuli may be justified when the research question requires context-specific materials, such as brands, products, interfaces, advertisements, destinations, or consumption scenarios.

This distinction can be observed in published neuromarketing studies. For example, when the aim is to analyze responses to audiovisual commercial stimuli involving the manipulation of product–voice congruence, as in Casado-Aranda et al. (2018), the use of specific stimuli may be justified by the need to represent realistic advertising configurations. Similarly, studies on word-of-mouth communication following product crises, such as Hsu and Cheng (2018), or on trust cues in online environments, such as Hubert et al. (2018), require stimuli tailored to the experimental context because the mechanisms under investigation depend on communicative, commercial, or situational attributes that are difficult to capture using generic affective databases. However, precisely in these cases, the methodological justification for ad hoc stimuli must be accompanied by sufficient documentation to enable ethical evaluation, theoretical interpretation, and, to the extent possible, replication.

To make these requirements operational, Table 4 presents a checklist of the minimum information that should be reported when selecting and documenting stimuli in neuromarketing studies. The checklist is intended not as a rigid template, but as a practical reporting guide for assessing whether the use of standardized or ad hoc stimuli is theoretically justified, ethically transparent, reproducible, and compatible with responsible use of research resources.

Based on this checklist, the decision between standardized and ad hoc stimuli can be organized through a sequential logic. First, researchers should determine whether the research question concerns general affective or cognitive mechanisms. If so, standardized stimuli should be considered as the first option. Second, if the question requires the representation of a specific brand, destination, product, interface, advertising message, or consumption situation, the use of ad hoc stimuli may be justified. Third, when ad hoc stimuli are used, researchers should document their affective, perceptual, and contextual properties through pretesting or prior validation. Finally, if the stimuli cannot be shared due to copyright or licensing restrictions, a sufficiently detailed description should be provided to enable critical evaluation and, where possible, the conceptual reproduction of the design.

In summary, the proposed decision logic can be condensed into an operational rule: use standardized stimuli when the aim is to study general affective or cognitive mechanisms; employ ad hoc stimuli when the research question requires the representation of a specific brand, product, interface, message, or consumption context; and, in both cases, document the properties, validation procedures, availability conditions, and potential ethical or legal restrictions of the stimuli.

### 8.6. Generative AI and the Future of Affective Stimulus Generation

A recent development that further reinforces the relevance of the proposed framework is the use of generative artificial intelligence (AI) to create emotionally evocative experimental stimuli. Generative AI expands the range of possible materials by enabling researchers to produce customized images, advertising messages, interfaces, product scenarios, brand-related content, and consumption contexts. In advertising research, for example, the MADE framework has been proposed as a structured approach for creating effective experimental stimuli using generative AI, emphasizing methodological rigor, empirical validation, and transparency in AI-assisted stimulus development (van Berlo et al., 2024). Similarly, recent work in affective science suggests that AI-generated images may elicit emotional responses comparable to those produced by existing affective databases when they are properly validated (Chiu et al., 2026).

However, the use of generative AI does not eliminate the methodological and ethical concerns addressed in the present framework; rather, it introduces a new context in which these concerns become especially relevant. AI-generated stimuli should not be treated as inherently valid, standardized, or replicable merely because they are realistic, customizable, or easy to produce. At a minimum, researchers should report the AI system or model employed, the prompt structure, the generation procedure, the criteria used to select or exclude outputs, any manual editing, the validation results, copyright or licensing conditions, and potential restrictions on material sharing (van Berlo et al., 2024).

This level of documentation is particularly important because AI-generated materials may contain features that are not immediately evident to the researcher but may nevertheless influence participants’ responses. These include visual artifacts, unintended affective cues, culturally specific meanings, demographic biases, stereotypical representations, or uncontrolled perceptual properties. Such risks are especially relevant in neuromarketing, where subtle variations in visual, emotional, or contextual features may affect psychophysiological or neural responses (Bradley & Lang, 1994; Karmarkar & Plassmann, 2019). For this reason, AI-generated stimuli should be subjected to systematic pretesting or validation procedures, particularly when they are used to elicit emotional responses, simulate commercial contexts, or represent specific social groups, brands, products, or consumption scenarios (Chiu et al., 2026; van Berlo et al., 2024).

Accordingly, generative AI should be understood as a specific form of ad hoc stimulus generation. Its use may be methodologically justified when the research question requires context-specific or commercially realistic materials that cannot be obtained from existing standardized databases (van Berlo et al., 2024). Nevertheless, from the perspective of the proposed framework, such use should be evaluated according to the same four principles: ethical transparency, scientific responsibility and replicability, sustainability, and participant protection. Within this logic, standardized affective databases should remain the preferred option when the aim is to examine general affective or cognitive mechanisms, whereas AI-generated stimuli may be justified when the study requires the representation of specific brands, products, platforms, messages, or consumption contexts (Chiu et al., 2026; Lang et al., 2008).

### 8.7. Integration of the Normative Framework

Based on the principles and operational criteria outlined above, the proposed framework defines an integrated logic for the responsible selection of stimuli in neuromarketing. Rather than prohibiting the use of context-specific or brand-related stimuli, the framework repositions them within a hierarchical methodological logic in which standardization serves as the default starting point and customization requires explicit theoretical or ap-plied justification. This logic is consistent with broader principles of research transparency, reproducibility, responsible innovation, and reusable research materials, while adapting them to the specific problem of stimulus selection in neuromarketing (Nosek et al., 2015; Wilkinson et al., 2016).

From this perspective, standardized affective databases can be understood as being simultaneously aligned with ethical transparency, scientific responsibility, sustainability, and participant protection. By contrast, the use of ad hoc stimuli, including AI-generated stimuli, should be interpreted as a conditional methodological strategy, justified in terms of its added theoretical or contextual value. This is especially important because generative AI can facilitate the creation of realistic experimental stimuli, but such stimuli still require transparent documentation, validation, and ethical assessment before being treat-ed as scientifically reliable materials (Chiu et al., 2026; van Berlo et al., 2024).

Beyond its practical implications, this framework contributes to the conceptual development of neuromarketing by establishing stimulus selection as a central domain of normative and epistemic regulation. It also provides a shared reference structure that can facilitate dialogue among researchers, practitioners, and ethics committees, supporting the advancement of more transparent, responsible, and sustainable neuromarketing practices (Murphy et al., 2008; Plassmann et al., 2012; Stanton et al., 2017). This is especially important because generative AI can facilitate the creation of realistic experimental stimuli, but such stimuli still require transparent documentation, validation, and ethical assessment before being treated as scientifically reliable materials (van Berlo et al., 2024; Chiu et al., 2026).

## 9. Discussion

The normative framework proposed in this article has implications for both academic research and applied neuromarketing practice. Its contribution is not to claim that stimulus selection has the same centrality across all neuromarketing designs, but rather to show that its relevance increases when stimuli constitute the main manipulation of the study or when their affective, perceptual, or contextual properties shape the interpretation of the observed neurophysiological responses. From this perspective, stimulus selection should be understood as a methodological decision with ethical, epistemic, and sustainability implications, especially in studies focused on emotion, attention, trust, reward, brand responses, or the evaluation of persuasive messages (Christensen et al., 2022; Plassmann et al., 2015).

From a conceptual perspective, the contribution of the framework is not to replace broader frameworks on research ethics, open science, or responsible research conduct, but rather to translate these principles into a specific methodological decision that often receives less explicit attention in neuromarketing: stimulus selection (Nosek et al., 2015; Owen et al., 2012; Stilgoe et al., 2013; Wilkinson et al., 2016). In doing so, the study turns the choice between standardized and ad hoc stimuli into a point of articulation between methodological validity, transparency, replicability, sustainability, and participant welfare.

### 9.1. Implications for Academic Research

For academic research, this work highlights stimulus selection as a core component of experimental design rather than a secondary procedural decision. While prior methodological discussions in neuromarketing have predominantly focused on neuroscientific techniques and data analysis, the present framework shifts attention toward the role of stimuli as the primary interface through which affective processes are elicited and measured (Karmarkar & Yoon, 2016; Poels & Dewitte, 2006).

A more systematic use of standardized affective stimuli can enhance replicability and cross-study comparability, thereby facilitating cumulative knowledge development and enabling more robust integration of findings in systematic reviews and meta-analyses (Branco et al., 2023). This is particularly relevant for a field seeking to consolidate its scientific foundations and strengthen its interdisciplinary connections with psychology and affective neuroscience, overcoming the current “crisis of reproducibility” (Balconi & Sansone, 2021).

In addition, the adoption of explicit criteria for transparency and justification in stimulus selection can contribute to raising methodological standards. Systematic reporting of affective properties and explicit justification of ad hoc stimulus construction promote greater clarity in experimental design and reinforce the credibility of empirical findings. As argued by Stanton et al. (2017), full transparency in protocols is one of the most effective ways to convey research competence and protect against the risks of overstating results.

The emphasis on sustainability further extends current methodological debates by introducing resource efficiency as a relevant dimension of research quality. Incorporating considerations of experimental redundancy and resource use into study design aligns neuromarketing research with broader institutional expectations regarding responsible and sustainable science (EFPA, 2025; IEA, 2025). This perspective acknowledges that scientific progress must minimize its environmental footprint while optimizing its discovery potential (Christensen et al., 2022).

From a sustainability perspective, the findings of this review suggest that the development of ad hoc stimuli should be explicitly justified when it involves additional stages of instrumental validation. This recommendation does not imply rejecting ad hoc stimuli, which may be necessary for reasons of ecological validity, contextual specificity, or alignment with the research question. However, when previously validated normative materials are available, appropriate for the study objective, and compatible with the target population, their reuse may reduce the need for additional instrumental piloting, improve comparability across studies, and promote more efficient and reproducible research. This proposal is consistent with broader calls to increase research value and reduce waste by avoiding unnecessary data collection, improving documentation, and reusing existing resources when scientifically appropriate (Ioannidis et al., 2014).

Finally, this work identifies the development of marketing-relevant affective databases as a key research agenda. Creating repositories that combine standardized affective metrics with contextual relevance to consumer behavior would enable greater methodological rigor without sacrificing ecological validity (Yang et al., 2018). Such initiatives would provide reliable corpus material for the scientific community, encouraging direct comparisons between research outcomes and enhancing the long-term credibility of the field.

### 9.2. Implications for Business and Professional Practice

From an applied perspective, the proposed framework provides guidance for companies, consultancies, and practitioners employing neuromarketing techniques in commercial contexts. Although the use of ad hoc stimuli is often justified by the need to work with brand-specific materials, an exclusive reliance on customized stimuli may introduce avoidable costs, methodological limitations, and ethical risks (Ariely & Berns, 2010; Stanton et al., 2017). In an industry where many techniques remain in their infancy, adopting standardized protocols is essential to facilitate the confident application of these tools to marketing problems (Ohme et al., 2009).

The strategic use of standardized affective stimuli in exploratory or early-stage research can improve efficiency by reducing the need for repeated pretesting and enabling more focused subsequent application to brand-specific materials (Branco et al., 2023; Lang et al., 2008). This staged approach allows organizations to combine methodological rigor with contextual relevance, minimizing the time- and resource-consuming task of running independent validation studies for every new project (Branco et al., 2023). Furthermore, the use of wearable and portable technologies, such as wireless EEG, can further enhance this efficiency in naturalistic commercial settings (Stanton et al., 2017; Wang et al., 2024).

Moreover, greater transparency in stimulus selection can strengthen the ethical legitimacy of neuromarketing practices. In a context of increasing public and regulatory scrutiny of persuasive technologies (Christensen et al., 2022; Ferrell et al., 2025), the ability to demonstrate adherence to principles of transparency, accountability, and participant protection becomes a critical factor for the long-term sustainability of the field. As industry practices are often perceived as proprietary and opaque, adopting open standards for stimulus characterization can mitigate fears of “mind control” and “stealth marketing” (Murphy et al., 2008; Stanton et al., 2017).

The framework may also serve as a common reference for communication among researchers, practitioners, clients, and ethics committees, facilitating the evaluation and justification of neuromarketing research designs (Hensel et al., 2017; Pop et al., 2014). Adhering to established guidelines, such as the NMSBA Code of Ethics, provides a necessary foundation for professional integrity and helps manage the “seductive” appeal of neuroscientific findings by ensuring they are grounded in valid scientific principles (Murphy et al., 2008; NMSBA, 2013). In this way, the contribution extends beyond individual studies to support the development of more consistent, transparent, and responsible professional standards across the neuromarketing industry (Stanton et al., 2017).

## 10. Conclusions

This paper has examined the role of affective stimulus selection in neuromarketing as a key dimension of responsible research practice. Through an integrated conceptual and normative analysis, it highlights how the choice between ad hoc and standardized stimuli carries implications for ethical transparency, epistemic robustness, sustainability, and participant welfare.

The main contribution of this article is to reframe stimulus selection as a methodological decision that should be justified according to the purpose of the research. The proposed framework does not argue that standardized stimuli are universally superior or that ad hoc stimuli should be avoided. Rather, it advances a conditional logic: standardized stimuli are especially useful when the aim is to study general affective or cognitive mechanisms, whereas ad hoc stimuli may be necessary when the research question depends on specific brands, products, interfaces, messages, destinations, or consumption contexts.

From this perspective, the framework helps clarify the conditions under which each strategy can be considered methodologically responsible. It also provides operational criteria for documenting the origin of stimuli, their affective properties, validation procedures, availability, legal restrictions, and potential implications for participants. In this way, stimulus selection becomes a point of articulation between methodological validity, reproducibility, sustainability, and research ethics.

This work is not without limitations. First, the sustainability analysis is based on illustrative scenarios and order-of-magnitude estimates, rather than on direct empirical measurements of energy consumption and emissions in specific laboratories. Second, the parameters used to estimate the consumption associated with fMRI validation stages—sample size, session duration, and the general structure of the protocol—are drawn from neuroimaging and cognitive neuroscience studies, since the neuromarketing literature rarely reports these data systematically for pretesting or instrumental validation stages (Dougherty et al., 2015; Leeds & Tarr, 2016; Mumford & Nichols, 2008; Poldrack et al., 2008). Although these values are consistent with standard fMRI protocols, their extrapolation to the neuromarketing context introduces uncertainty. Third, the proposed framework is conceptual and requires empirical validation across different cultural, technological, institutional, and applied contexts.

These limitations open several avenues for future research. Experimental studies could directly compare standardized stimuli, ad hoc stimuli, and mixed sequential designs in terms of internal validity, ecological validity, affective intensity, neurophysiological responses, replicability, and validation costs. Content analyses could also apply the proposed checklist to published studies in order to assess the degree of transparency in stimulus reporting. Delphi studies involving researchers, practitioners, and ethics committees would also be useful for evaluating the applicability of the framework and refining its criteria. Finally, empirical laboratory audits could directly measure energy consumption, instrumental use time, pilot sessions, repetitions, and discarded materials associated with different stimulus selection strategies.

Taken together, this article contributes to advancing a more transparent, methodologically robust, and sustainable form of neuromarketing. By highlighting that stimulus selection is not merely a procedural decision, but a practice with scientific, ethical, and environmental consequences, this work invites researchers and practitioners to reassess established practices and to adopt more explicit, documented, and responsible criteria in the design of neuromarketing studies.

## Figures and Tables

**Table 1:** Comparative synthesis of standardized and ad hoc stimuli in neuromarketing.

Dimension of Analysis	Standardized Stimuli	Ad Hoc Stimuli
Experimental control	Advantage: High control over affective dimensions and physical properties (Bradley & Lang, 1994; De Cesarei & Codispoti, 2006; Gerges et al., 2025).	Limitation: Risk of confounding variables, such as brand, music, or design features, which make it difficult to isolate psychological mechanisms (Ariely & Berns, 2010; Lim, 2018).
Ecological validity	Limitation: Restricted; isolated materials that do not reproduce the complexity of real consumption contexts (Bilucaglia et al., 2024; Gerges et al., 2025).	Advantage: High; they incorporate real market elements, such as visual identity and narrative, as well as commercial environments (Ariely & Berns, 2010; Lim, 2018)
Replicability and comparability	Advantage: Facilitate the accumulation of evidence and comparisons across laboratories (Kurdi et al., 2017; Lindquist et al., 2012).	Limitation: Difficult when stimuli are unique, inaccessible, or protected by copyright (Stanton et al., 2017; Wang et al., 2024).
Relevance for marketing	Limitation: They do not always correspond to constructs such as loyalty or purchase intention (Casado-Aranda et al., 2023; Cherubino et al., 2019).	Advantage: Maximum correspondence with the consumption phenomenon and applied constructs (Alsharif et al., 2021; Plassmann et al., 2012).
Representativeness	Limitation: Cultural and demographic biases, including WEIRD samples, and material obsolescence (Gerges et al., 2025; Lang et al., 2008).	Advantage: High flexibility to adapt the design to specific populations and sectors (Alsharif et al., 2021; Plassmann et al., 2012).
Resources and validation	Advantage: Effective and validated starting point that supports the justification of stimulus selection to reviewers (Casado-Aranda et al., 2023; Cherubino et al., 2019).	Limitation: High experimental burden; they require rigorous pretesting, greater funding, and more time (Cenizo, 2022; Plassmann et al., 2012).
Ethical considerations	Documented history that allows researchers to anticipate effects and minimize harm (Lang et al., 2008; Senior & Lee, 2008).	Limitation: Risk of unconscious influence and lack of prior documentation to anticipate adverse effects (Ariely & Berns, 2010; Ferrell et al., 2025).

**Table 2:** Representative affective resources and related datasets relevant to neuromarketing.

Database/Set	Main Stimulus Type	Resource Type	Reference	Normative Data Provided	Typical Uses
IAPS	Static color images	Normed affective stimulus database	(Lang et al., 2008)	Valence, arousal, dominance (SAM)	EEG/ERP, EDA, fMRI, behavioral research
ANEW	Words	Normed affective word database	(Bradley & Lang, 1999)	Valence, arousal, dominance (SAM)	Language, memory, decision making
IADS/IADS-E	Natural sounds (6 s)	Normed affective auditory database	(Yang et al., 2018)	Valence, arousal, dominance (SAM)	Auditory emotion, sensory interaction
DEAP	Music videos (1 min)	Multimodal psychophysiological response dataset	(Koelstra et al., 2012)	Valence, arousal, dominance, liking, familiarity	Multimodal emotion analysis, EEG/physiological signals
DREAMER	Movie clips (65–393 s)	Psychophysiological response dataset	(Katsigiannis & Ramzan, 2018)	Valence, arousal, dominance	Emotion recognition through wireless low-cost EEG/ECG
EmoStim	Video clips (avg. 133 s)	Dynamic affective stimulus set	(Somarathna et al., 2024)	14 discrete emotions and 39 CPM descriptors	Dynamic emotion induction, affective computing
EmoEEG-MC	Videos and imagery tasks	Psychophysiological/neural response dataset	(Xu et al., 2025)	64-channel EEG, GSR, PPG, 7 labels	Cross-context emotion decoding, neural substrate analysis

Note: EEG = electroencephalography; ERP = event-related potentials; EDA = electrodermal activity; fMRI = functional magnetic resonance imaging; ECG = electrocardiography; GSR = galvanic skin response; Pos/Neu/Neg = positive/neutral/negative; PPG = photoplethysmography; Temp = body temperature; CPM descriptors = component process model descriptors. The table presents a representative selection of affective resources and related datasets, drawn from the broader classification matrix reported in Supplementary Table S1: Bibliographic dataset of standardized stimulus resources, rather than an exhaustive inventory. Resource type was assigned according to the primary function of each resource: provision of normed stimuli, dynamic affective induction, psychophysiological or neural response recording, or computational emotion analysis. The categories are intended to clarify functional differences between resources, not to imply that all datasets are directly interchangeable as standardized stimulus sets. References in the “Reference” column correspond to the primary normative or dataset descriptions of each resource.

**Table 3:** Illustrative estimation of energy consumption in neuromarketing experimental sessions.

Technique	Equipment and Operational Assumption	Estimated Consumption	Estimated Emissions	Scope
fMRI, low scenario	Siemens MAGNETOM Trio/Trio Tim; 12 participants × 1.5 h; standby, 13 kW-equivalent	234 kWh	46.8–58.5 kgCO_2_e	Lower operational bound; does not represent full functional acquisition
fMRI, high scenario	Siemens Siemens MAGNETOM Trio/Trio Tim; 12 participants × 1.5 h; maximum average power, 54 kW-equivalent	972 kWh	194.4–243.0 kgCO_2_e	High-demand scenario; not equivalent to a real session measurement
EEG	BioSemi ActiveTwo Mk2; 16 participants × 50.7 min; amplifier, 4 W	0.054 kWh	10.8–13.5 gCO_2_e	Amplifier only during effective recording time; excludes computer, monitors, laboratory infrastructure, and consumables

Note: EEG = electroencephalography; fMRI = functional magnetic resonance imaging. Emissions were calculated by applying an illustrative range of 0.20–0.25 kgCO_2_e/kWh, based on European metrics of electricity emissions intensity (European Environment Agency, 2025). The actual value depends on the country, year, local electricity mix, and specific laboratory infrastructure. The fMRI values are derived from specifications expressed in kVA; to facilitate estimation, they are expressed as kW-equivalent under the conservative assumption of a power factor equal to 1. Therefore, the results should be interpreted as illustrative scenarios rather than empirical measurements of actual electricity consumption. The fMRI values are taken from the technical datasheet of the Siemens Trio A Tim System (Siemens Medical Solutions, 2026).

**Table 4:** Checklist for stimulus selection and reporting in neuromarketing studies.

Dimension	Minimum Information to Be Reported	Practical Application
Stimulus source	Indicate whether the stimulus comes from a standardized database, open repository, existing commercial material, or ad hoc creation	Enables assessment of traceability and reproducibility
Theoretical justification	Explain which construct the stimulus activates or represents: valence, arousal, trust, congruence, threat, reward, etc.	Prevents stimulus selection from appearing arbitrary
Affective properties	Report normative values or pretest results for valence, arousal, dominance, familiarity, or relevance	Necessary for interpreting psychophysiological or neural responses
Contextual appropriateness	Justify why the stimulus is appropriate for the phenomenon under study, especially in relation to brands, products, tourism, advertising, or digital commerce	Justifies the use of ad hoc stimuli when standardized databases are insufficient
Pretesting procedure	Describe the sample, sample size, scales, exclusion criteria, manipulation, and main results	Enables assessment of the robustness of prior validation
Physical characteristics	Report duration, resolution, brightness, volume, size, language, format, number of stimuli, and experimental conditions	Controls for potential perceptual confounds
Material availability	Indicate whether the stimuli are available in a repository, upon request, or restricted by copyright	Facilitates replication and transparency
Rights and licenses	Specify copyright status, permissions for use, commercial restrictions, or inability to share the material	Addresses ethical and legal requirements
Participant risk	Indicate whether the stimuli are emotionally intense, sensitive, potentially aversive, or culturally delicate	Protects participant well-being
Validation cost	Report whether pilot testing, instrumental sessions, discarded stimuli/sessions, or additional calibrations were involved	Connects stimulus selection with sustainability and resource efficiency

## Data Availability

The data presented in this study are openly available in the Open Science Framework (OSF) at https://doi.org/10.17605/OSF.IO/KMFBS under the title Bibliographic dataset of standardized stimulus resources. The repository contains the full dataset in Excel and CSV formats (bibliographic_dataset_of_standardized_stimulus_v1.0_2026.xlsx and bibliographic_dataset_of_standardized_stimulus_v1.0_2026.csv), from which Table 2 in the main manuscript and Supplementary Table S1 were derived.

## Supplementary Materials

The following supporting information can be downloaded at: https://www.mdpi.com/article/10.3390/bs16071115/s1, Table S1: Bibliographic dataset of standardized stimulus resources.

## Abbreviations

EEG	electroencephalography
ERPs	event-related potentials
EDA	electrodermal activity
fMRI	functional magnetic resonance imaging
ECG	electrocardiography
GSR	galvanic skin response
Pos	positive
Neu	neutral
Neg	negative
PPG	photoplethysmography
Temp	body temperature
CPM descriptors	component process model descriptors
