# Supplementary material for: Responsible Stimulus Selection in Neuromarketing: A Critical Narrative Review and Normative Framework for Ethical, Sustainable, and Replicable Consumer Research
Source: Behav Sci (Basel). 2026 Jul 3;16(7):1115. doi: 10.3390/bs16071115 (PMC13403436; doi:10.3390/bs16071115)
Supplement: Supplementary file 1 [file behavsci-16-01115-s001.zip › behavsci-4325391-supplementary.pdf]

Supplementary Material

Responsible stimulus selection in neuromarketing: A critical narrative review and normative framework for ethical, sustainable, and replicable consumer research

Alberto Ruiz-Osta [1,*], Casandra I. Montoro [2] and Eduard Cristobal-Fransi [1]

[1] Department of Business and Economics, Faculty of Law, Economics and Tourism, University of Lleida, 25001 Lleida, Spain; eduard.cristobal@udl.cat
[2] Department of Psychology, University of Jaén, 23071 Jaén, Spain; imontoro@ujaen.es
* Correspondence: aro10@alumnes.udl.cat

Supplementary Table S1. Bibliographic dataset of standardized stimulus resources.

**Resource name**	**Year**	**DOI**	**Resource type**	**Stimulus modality**	**Affective norms or ratings**	**Rating scales**	**Physiological or neural data**
COVEE	2026	10.1016/j.neucom.2025.132167	Psychophysiological response dataset	Multimodal; Physiological data; Eye-tracking; Video	cognitive load; situation awareness; affective components	NASA-TLX; ISA	EEG; eye-tracking
FACEMORPHIC	2026	10.1016/j.cviu.2025.104578	Emotion-recognition dataset	Video; Other (Event data); Faces	Action Unit intensities; valence; arousal	dimensional ratings; FACS AUs	none
not specified	2026	10.1016/j.iswa.2026.200636	Domain-specific stimulus set	Video; Audio; Faces; Eye-tracking	deception state; FACS AUs	binary ratings; Action Units	none
not specified	2026	10.1016/j.applanim.2026.107035	Emotion-recognition dataset	Faces, Images (Horse)	pain scores (HGS)	grimace scale	none
CAS-AIR-3D	2026	10.1007/s11263-025-02674-2	Domain-specific stimulus set	Faces	identities, expressions	not reported	none
FLAME	2026	10.1109/taffc.2026.3679039	Emotion-recognition dataset	Faces	discrete emotions	not reported	none
not specified	2026	10.3758/s13428-026-03010-3	Normed affective stimulus database	Text	valence, arousal, discrete emotions (10)	5-point Likert	none

Figurative Archive	2026	10.1038/s41597-025-06459-7	Normed affective stimulus database	Text	familiarity, concreteness, meaningfulness	7-point Likert	none
EmoWork	2026	10.1038/s41597-025-06531-2	Psychophysiological response dataset	Multimodal	stress, arousal, valence, emotional workload	Likert	EEG, ECG, BVP, EDA, video features
PEGCONV (Extended)	2026	10.1038/s41597-026-06819-x	Psychophysiological response dataset	Multimodal	speech patterns, gaze, gestures	not reported	motion capture, gaze tracking
not specified	2026	10.1038/s41597-026-07098-2	Dynamic/multimodal stimulus set	Faces	discrete emotions (6), AU labels	not reported	FACS
SMSAT	2026	10.1109/taffc.2026.3653301	Psychophysiological response dataset	Audio	physiological responses	physiological indicators	ECG/HR/HRV
RTT	2026	10.1016/j.dsp.2025.105470	Emotion-recognition dataset	Video	not specified	not reported	none
NEVi	2026	10.1038/s41597-026-06870-8	Normed affective stimulus database	Video	valence, arousal	dimensional ratings	none
SCED (Sound-based community emotion detection) Dataset	2026	10.1016/j.bspc.2026.109534	Normed affective stimulus database	Audio	naming accuracy, familiarity, pleasantness	Likert	none
AffectNet+	2026	10.1109/taffc.2025.3634523	Emotion-recognition dataset	Faces; Images	valence; arousal; discrete	dimensional ratings; discrete emotion ratings	FACS

					emotions; soft-labels		
IEMOCAP (personality extension)	2026	10.1109/taffc.2025.3637088	Normative adaptation/translation	Multimodal; Speech; Faces; Text	self-report ratings	dimensional ratings	FACS
CAST-Phys	2026	10.1109/taffc.2026.3678698	Psychophysiological response dataset	Video; Physiological data	valence; arousal	SAM	EDA/GSR; respiration; remote PPG
CogniFuse	2026	10.1109/tbme.2026.3691190	Psychophysiological response dataset	Physiological data	physiological responses	physiological indicators	EEG; EDA/GSR; PPG; respiration
Not specified	2026	10.1145/3776569	Emotion-recognition dataset	Multimodal; Visual; Audio; Text	intensity; discrete emotions	categorical indicators	none
not specified	2026	10.1109/taffc.2025.3650482	Psychophysiological response dataset	Physiological data; Video	valence; arousal; discrete emotions	self-report ratings	EEG
CleanFER25_RAF_CK	2026	10.3390/app16094415	Normative adaptation/translation	Faces; Images	discrete emotions	not reported	none
MM-MoralBench	2026	10.1016/j.patcog.2026.113624	Domain-specific stimulus set	Images; Text	discrete emotions (moral judgments)	discrete emotion ratings	none
MFEA-Bench	2026	10.1016/j.patcog.2026.113519	Emotion-recognition dataset	Faces	discrete emotions, intensity	discrete emotion ratings	none

MSE-LAM	2026	10.1016/j.neucom.2025.132279	Emotion-recognition dataset	Faces	discrete emotions	discrete emotion ratings	none
E-DLL	2026	10.1016/j.ijchp.2026.100690	Psychophysiological response dataset	Video	auto-reporte emoción/intensidad, traza de valencia	FeelTrace, Likert (0-9)	none
Not specified	2026	10.1016/b978-0-443-29139-5.00004-5	Normative adaptation/translation	Faces	intensidad, valencia, arousal, precisión	Likert 1-9, tarea 7 opciones	none
MCCCD	2026	10.1109/tpami.2025.3603653	Dynamic/multimodal stimulus set	Faces, Video	discrete emotions, intensity	categorical labels, Likert	none
OPEN_EmoRec_II	2026	10.4324/9781003582861-8	Psychophysiological response dataset	Multimodal	valence, arousal, dominance	SAM, labels	physiology
not specified	2025	10.1016/j.cag.2025.104455	Emotion-recognition dataset	Multimodal; Video; Eye-tracking; Audio	discrete emotions; personality traits	dimensional ratings (Big Five); FACS AUs	none
HBUED	2025	10.1016/j.jad.2025.119397	Psychophysiological response dataset	Video; Physiological data	valence; arousal	SAM; dimensional ratings; Likert	EEG; ECG; EDA/GSR
OffVA	2025	10.1016/j.csl.2025.101781	Domain-specific stimulus set	Images; Text	valence; arousal	dimensional ratings; Best-Worst-Scaling (BWS)	none

M2-S2ETH	2025	10.1016/j.knosys.2025.113995	Dynamic/multimodal stimulus set	Audio; Text; Speech	discrete emotions; sentiment; humor; sarcasm; toxicity	discrete emotion ratings; Likert	none
ZJXU-MOTION	2025	10.1016/j.bspc.2024.107445	Psychophysiological response dataset	Video; Physiological data; Faces	physiological responses	physiological indicators	Heart rate; BVP
CN-VEFD	2025	10.1016/j.dib.2025.112305	Domain-specific stimulus set	Images; Facial expression data	color emotion; composition emotion; facial affect	dimensional ratings (valence); discrete categories	none
EmoWear	2025	10.1109/taffc.2025.3575281	Psychophysiological response dataset	Video; Physiological data; Other	valence; arousal	dimensional ratings; SAM	ECG; BVP; RSP; EDA; SKT; SCG
Facial-BSL	2025	10.13053/cys-29-2-5119	Domain-specific stimulus set	Video; Faces	discrete emotions (sign-related)	discrete emotion ratings	none
Italian Chat Dataset	2025	10.1145/3712260	Domain-specific stimulus set	Text	impulsiveness; psychological factors; self-report	Likert; BIS-11; IRI	none
REELMO	2025	10.1038/s41597-025-05159-6	Psychophysiological response dataset	Multimodal	20 affective states, overall liking	continuous real-time reports	fMRI
not specified	2025	10.1111/exsy.13832	Normed affective stimulus database	Text	discrete emotions	expert annotations	none

PARSNiP	2025	10.1007/s12369-025-01266-x	Psychophysiological response dataset	Multimodal	valence, arousal, attention	7-point scale	none
not specified	2025	10.3389/fpsyg.2025.1454312	Normed affective stimulus database	Faces	age, gender, ethnicity, emotion	discrete judgments	none
UpStory	2025	10.3389/frobt.2025.1547578	Emotion-recognition dataset	Multimodal	valence, arousal, dominance, rapport	5-point SAM	motion capture, facial expressions
not specified	2025	10.3390/info16070562	Normative adaptation/translation	Words	valence	SAM (1-9)	none
German word-picture database	2025	10.1371/journal.pone.0336981	Normed affective stimulus database	Multimodal	valence, arousal, familiarity, action relatedness	1-7 Likert	none
not specified	2025	10.1016/j.bspc.2025.107632	Emotion-recognition dataset	Multimodal	discrete emotions	expert annotations	none
PROPER	2025	10.1109/access.2024.3395434	Psychophysiological response dataset	Physiological data	self-report ratings	Big Five Personality Trait questionnaire	EEG
Navarasa	2025	10.1007/s00371-025-03911-y	Emotion-recognition dataset	Faces	discrete emotions	etiquetas categóricas	none

OYH	2025	10.1007/s10586-025-05830-y	Emotion-recognition dataset	Speech	discrete emotions, self-report ratings	Not reported	none
VAMOS	2025	10.3758/s13428-024-02510-4	Normed affective stimulus database	Images	valence, arousal, intensity, memorability	9-point Likert	none
VISIONS	2025	10.3758/s13428-024-02535-9	Normed affective stimulus database	Images	familiarity, visual complexity, clarity	eye-tracking	eye-tracking
ELI	2025	10.3758/s13428-024-02585-z	Domain-specific stimulus set	Video	identification accuracy	not reported	none
IFD	2025	10.3758/s13428-025-02723-1	Normed affective stimulus database	Faces	valence, discrete emotions, social ratings	VAS	none
ECBD	2025	10.3758/s13428-025-02766-4	Dynamic/multimodal stimulus set	Multimodal	tear intensity, vocalizations, sadness	7-point Likert	none
COD	2025	10.3758/s13428-025-02849-2	Normed affective stimulus database	Text	valence, arousal, familiarity, intensity	Likert	none
CEPAV	2025	10.1038/s41597-024-04364-z	Psychophysiological response dataset	Multimodal	discrete emotions, stress, appraisals	7-point Likert	ECG, EDA, BP, respiration
TCP	2025	10.1038/s41597-025-04895-z	Psychophysiological response dataset	Physiological data	pleassure, emotion regulation, symptoms	psych assessments	fMRI

Spacetop	2025	10.1038/s41597-025-05154-x	Psychophysiological response dataset	Multimodal	emotional responses, valence	gLMS, Likert	fMRI, EDA, PPG
EmoEEG-MC	2025	10.1038/s41597-025-05349-2	Psychophysiological response dataset	Multimodal	valence, arousal, discrete emotions, liking	continuous scale	EEG, GSR, PPG
(Aging Faces)	2025	10.1038/s41597-025-05909-6	Normed affective stimulus database	Faces	authenticity, age norms	not reported	none
not specified	2025	10.1007/s11227-025-07273-z	Psychophysiological response dataset	Physiological data	behavioural responses	not reported	none
CHILL	2025	10.1038/s41746-025-02192-y	Psychophysiological response dataset	Video	physiological responses (HR)	not reported	PPG
VREEG	2025	10.1016/j.bspc.2025.107674	Psychophysiological response dataset	Physiological data	discrete emotions, self-report ratings	self rating scale	EEG
THERADIA WoZ	2025	10.1109/taffc.2025.3557465	Emotion-recognition dataset	Multimodal	valence, arousal, discrete emotions, intensity, novelty, intrinsic pleasantness, goal conduciveness, coping	dimensional ratings, discrete emotion ratings	none
MESC	2025	10.1109/tmm.2025.3604951	Emotion-recognition dataset	Multimodal	discrete emotions, self-report ratings	discrete emotion ratings	none

not specified	2025	10.1371/journal.pone.0329554	Normed affective stimulus database	Faces	discrete emotions	forced-choice	none
not specified	2025	10.7717/peerj-cs.2676	Dynamic/multimodal stimulus set	Video (NIR/Visible)	discrete emotions	not reported	none
SEED-VII	2025	10.1109/taffc.2024.3485057	Normed affective stimulus database	Video	arousal, discrete emotions	PANAS, discrete emotion ratings	none
UniC	2025	10.1007/s10579-025-09837-0	Psychophysiological response dataset	Multimodal	valence, arousal, dominance, aggression, fear	dimensional ratings	ECG, GSR, EMG, motion capture
COST 2102	2025	10.3390/brainsci15030222	Dynamic/multimodal stimulus set	Multimodal	discrete emotions	not reported	none
MyMSC corpus	2025	10.1145/3703445	Emotion-recognition dataset	Multimodal; Video; Audio; Text	valence; discrete emotions; behavioural responses	not reported	none
SenseSeek Dataset	2025	10.1145/3749501	Psychophysiological response dataset	Multimodal; Physiological data; Eye-tracking	familiarity; relevance; physiological responses; behavioural responses	Likert; physiological indicators	EEG, EDA, eye-tracking, pupillometry, motion capture
REVIT	2025	10.1145/3770635	Psychophysiological response dataset	Video; Physiological data	physiological responses	physiological indicators	ECG, EEG, respiration
Affective Audio Dataset (AAD)	2025	10.1109/taffc.2024.3437153	Normed affective stimulus database	Audio	valence; arousal	dimensional ratings	none

						(Affective Slider)	
WIC640	2025	10.1186/s40708-025-00274-x	Domain-specific stimulus set	Images; Physiological data; Eye-tracking	valence; physiological responses	Likert	EEG; EDA/GSR; eye-tracking
BFER-Net	2025	10.1109/access.2025.3545759	Emotion-recognition dataset	Faces; Images	discrete emotions	not reported	none
DECEPTIcON	2025	10.1109/taffc.2025.3591205	Emotion-recognition dataset	Multimodal; Text; Audio; Video	credibility	discrete ratings (Truth-O-Meter)	none
EEGEmotions-27	2025	10.1109/access.2025.3620677	Psychophysiological response dataset	Physiological data; Video	discrete emotions (27 categories)	Likert	EEG
KIDO	2025	10.1109/access.2025.3606359	Normed affective stimulus database	Other (Drawings); Text	discrete emotions	not reported	none
Multi-HM	2025	10.3390/app15084509	Emotion-recognition dataset	Multimodal; Text; Audio; Video	valence; arousal; discrete emotions	not reported	none
AFTER	2025	10.3390/children12070816	Normed affective stimulus database	Faces; Images	discrete emotions	discrete emotion ratings	none
BER2024	2025	10.1016/j.compbiomed.2025.110350	Emotion-recognition dataset	Faces; Other (Body gestures)	discrete emotions	discrete emotion ratings	none
ADSIP database	2025	10.1109/access.2024.3420103	Dynamic/multimodal stimulus set	VR/AR	7 expresiones en tres intensidades	calificaciones de confianza	none

Through the Eyes of Emotion	2025	10.1145/3749545	Normative adaptation/translation	Faces	precisión de identificación (6 categorías)	selección de etiqueta	none
JNV corpus	2024	10.1016/j.specom.2023.103004	Normed affective stimulus database	Audio; Speech	discrete emotions; intensity; authenticity; realism	discrete emotion ratings; Likert	none
Spanish MEACorpus 2023	2024	10.1016/j.csi.2024.103856	Normed affective stimulus database	Audio; Text; Speech	discrete emotions	discrete emotion ratings (Ekman)	none
Tamil (MSAT)	2024	10.1016/j.asoc.2024.111553	Domain-specific stimulus set	Multimodal; Video; Audio; Text	sentiment; discrete emotions	discrete emotion ratings; 7-point Likert	none
VREMO	2024	10.1016/j.heliyon.2024.e38681	Psychophysiological response dataset	VR; Physiological data	valence; arousal; dominance	dimensional ratings (VAD); SAM	EEG
AF3dE	2024	10.1145/3631133	Domain-specific stimulus set	Images; Other (3D metadata)	discrete emotions; valence; arousal; dominance	dimensional ratings (VAD); discrete centers	none
PortraitEmotion3D	2024	10.3390/app142311235	Dynamic/multimodal stimulus set	Faces	discrete emotions, 3D (DVA)	dimensional ratings; discrete labels	none
ImaText	2024	10.1145/3589335.3651971	Emotion-recognition dataset	Multimodal	discrete emotions	not reported	none

InFER++	2024	10.1109/ojcs.2024.3443511	Emotion-recognition dataset	Faces	discrete emotions	discrete emotion labels	none
JVNV	2024	10.1109/access.2024.3360885	Normed affective stimulus database	Speech	discrete emotions, intensity	4-point intensity scale	none
VioMusic	2024	10.3390/info15040224	Normed affective stimulus database	Audio	valence, arousal	potency arousal model (1-5)	none
TUNA database	2024	10.1371/journal.pone.0302904	Normed affective stimulus database	Images	valence, arousal, anger, disgust, empathy, ambivalence	split semantic differential, Likert	none
MYFED	2024	10.1007/s00138-024-01625-0	Normed affective stimulus database	Video	discrete emotions	Not reported	FACS
ViMACSA	2024	10.1007/s00530-024-01558-8	Emotion-recognition dataset	Multimodal	self-report ratings	none (labels pos/neg/neu)	none
Mandarin Chinese Speech Database	2024	10.1007/s10579-024-09790-4	Domain-specific stimulus set	Speech	intensity, self-report ratings	Not reported	none
PD-Corpus	2024	10.1007/s13278-024-01219-8	Normed affective stimulus database	Text	discrete emotions	Not reported	none
EMMA	2024	10.3758/s13428-024-02336-0	Normed affective stimulus database	Audio	discrete emotions (GEMS), liking, familiarity	GEMS, Likert	none
KFS	2024	10.3758/s13428-024-02402-7	Normed affective stimulus database	Audio	valence, arousal, intensity,	Likert	none

					familiarity, appetizingness		
ECCS	2024	10.3758/s13428-024-02408-1	Normed affective stimulus database	Text	valence, arousal, discrete climate emotions	Likert	none
ICMR-Manipal	2024	10.3758/s13428-024-02439-8	Domain-specific stimulus set	Images	familiarity, name agreement	not reported	none
not specified	2024	10.3758/s13428-024-02444-x	Normed affective stimulus database	Images	valence, arousal, familiarity, clarity	Likert	none
DerLex	2024	10.3758/s13428-024-02565-3	Psychophysiological response dataset	Text	behavioural responses, valence (existing)	not reported	eye-tracking
NEMO	2024	10.1109/taffc.2023.3315971	Normed affective stimulus database	Images	familiarity, interest, appeal	7-point Likert	none
G-REx	2024	10.1038/s41597-023-02905-6	Psychophysiological response dataset	Physiological data	valence, arousal	SAM	PPG, EDA, HR
AVDOS-VR	2024	10.1038/s41597-024-02953-6	Psychophysiological response dataset	VR/AR	valence, arousal (continuous)	controller touchpad	PPG, EMG (facial), IMU
(Mixed Emotion)	2024	10.1038/s41597-024-03676-4	Psychophysiological response dataset	Multimodal	valence, arousal, discrete emotions	Likert, PANAS	EEG, GSR, PPG, face video
GameVibe	2024	10.1038/s41597-024-04022-4	Emotion-recognition dataset	Multimodal	engagement	affect traces	none

DEM	2024	10.1109/access.2024.3358207	Normed affective stimulus database	Faces	discrete emotions, intensity	discrete emotion ratings	none
Not specified	2024	10.1109/tbiom.2023.3324684	Normed affective stimulus database	Faces	discrete emotions	not reported	none
MelTrans	2024	10.3390/s24175506	Normed affective stimulus database	Speech	discrete emotions, intensity	5-point Likert	none
Travel Agency Task Dialogue Corpus	2024	10.1145/3675166	Dynamic/multimodal stimulus set	Multimodal; Video; Speech; Faces	discrete emotions; behavioural responses	physiological indicators	none
EAV	2024	10.1038/s41597-024-03838-4	Psychophysiological response dataset	Physiological data; Audio; Video	valence; arousal; discrete emotions	SAM; Likert	EEG
EmoStim	2024	10.1109/taffc.2023.3328900	Dynamic/multimodal stimulus set	Video	discrete emotions; intensity	Likert; CoreGRID	none
FBioT Dataset	2024	10.3390/electronics13244867	Emotion-recognition dataset	Video; Faces	discrete emotions	FACS-based indicators	FACS
BTFER	2024	10.1016/j.engappai.2024.108983	Emotion-recognition dataset	Faces	discrete emotions	discrete emotion ratings	none
MGEED	2024	10.1109/taffc.2023.3286351	Psychophysiological response dataset	Multimodal	valence, arousal, dominance, predictability	SAM	EEG, ECG, EDA, Resp, Eye-tracking

EmojiString	2024	10.24846/v33i1y202408	Normed affective database	Images	7 dimensions (valence, arousal, etc.)	Likert	none
SMIC	2024	10.1049/ipr2.13118	Psychophysiological response dataset	Video	positive, negative, surprise	self-report ratings	none
Chinese Norms	2024	10.3758/s13428-024-02437-w	Normed affective stimulus database	Words	valence, arousal, imagery	SAM/Likert	RTs
Persian Norms	2024	10.1007/s10936-024-10104-6	Normed affective stimulus database	Words	valence, arousal, animacy	not reported	none
TACO	2024	10.3758/s13428-024-02428-x	Domain-specific stimulus set	Words	valence, arousal, AoA	SAM/Likert	none
SNED	2024	10.3758/s13428-024-02556-4	Domain-specific stimulus set	Images	valence, arousal, mystery, interest, pleasantness, etc.	semantic slider (1-9)	none
EPPS	2024	10.3758/s13428-024-02418-z	Domain-specific stimulus set	Images	valence, arousal, dominance, disgust, moral acceptance	SAM, discrete scales	none
Tactile Interaction Dataset	2023	10.1145/3596258	Psychophysiological response dataset	Multimodal; Video; Other (Tactile)	valence; arousal; anxiety; attitude	dimensional ratings; Likert; SDS/SAS	none
NNC Dataset	2023	10.5334/jopd.93	Domain-specific stimulus set	Words; Text	interpretation data; interpretation time	lexical-semantic annotations	none

DCU Affect Dataset	2023	10.1007/s42514-023-00153-z	Psychophysiological response dataset	Multimodal; Video; Audio; Other (Depth)	arousal; valence; agreement; interest	dimensional ratings; continuous; Likert	none
ASED	2023	10.1145/3529759	Emotion-recognition dataset	Speech	discrete emotions	discrete emotion ratings	none
Mofa	2023	10.2139/ssrn.4326597	Psychophysiological response dataset	Multimodal; Speech	behavioural responses	physiological indicators	none reported in snippet
C2W2D	2023	10.3389/fpsyg.2022.1047427	Emotion-recognition dataset	Video	lying/truth-telling	FACS	FACS
EmoInt-Trans	2023	10.1109/taslp.2022.3224287	Emotion-recognition dataset	Multimodal	32 emotions, 15 empathetic intents	discrete emotion ratings	none
DAMI-P2C	2023	10.1109/taffc.2022.3178689	Emotion-recognition dataset	Multimodal	valence, arousal, engagement	valence-arousal scale	none
PhyMER	2023	10.1109/access.2023.3320053	Psychophysiological response dataset	Physiological data	discrete emotions	self-assessment	EEG, BVP
HAANet	2023	10.1109/tcss.2022.3187198	Emotion-recognition dataset	Video	ambiguous action classes	not reported	none
ATL-BP	2023	10.1109/tbiom.2022.3210479	Emotion-recognition dataset	Video, Facial expression data	problem outcomes	behavioural responses	none

UBFC-Phys	2023	10.1109/taffc.2021.3056960	UBFC-Phys	Psychophysiological response dataset	Video; Physiological data	self-reported anxiety, physiological responses	CSAI
EMAP	2023	10.1111/psyp.14446	Psychophysiological response dataset	Multimodal	arousal, valence, discrete emotions, liking	Likert, slider scale (0-10)	EEG, EDA, ECG, HR, respiration, blood volume
FeelingBlue	2023	10.1162/tacl_a_00540	Normed affective stimulus database	Images	discrete emotions, rationales	Best-Worst Scaling (BWS)	none
French word-association norms	2023	10.1177/17470218231154454	Normed affective stimulus database	Words	familiarity, concreteness, association	7-point scale	none
MET Corpus	2023	10.3390/jintelligence11070145	Normed affective stimulus database	Multimodal	discrete emotions, intensity, authenticity	multiple-choice, 5-point scales	none
PerceptSent	2023	10.1109/taffc.2022.3225238	Normed affective stimulus database	Images	valence, self-report ratings	Likert	none
IESC	2023	10.1007/s11042-023-14577-w	Domain-specific stimulus set	Speech	discrete emotions	discrete emotion ratings	none
EmoMatchSpanishDB	2023	10.1007/s11042-023-15959-w	Emotion-recognition dataset	Speech	discrete emotions	cuestionario de opción múltiple	none

DEKT-345 × 2	2023	10.1007/s11042-023-17653-3	Psychophysiological response dataset	Text	valence, discrete emotions	Not reported	none (keystroke dynamics)
CVFD	2023	10.3758/s13428-022-02047-4	Domain-specific stimulus set	Words	valence, self-report ratings	Not reported	none
EPSS	2023	10.3758/s13428-023-02087-4	Normed affective stimulus database	Multimodal	valence, arousal, dominance, intensity, self-report ratings	Likert	none
GAUDIE	2023	10.3758/s13428-023-02135-z	Normed affective stimulus database	Audio	valence, arousal, dominance, discrete emotions, familiarity, intensity	SAM, GEW, Likert	none
CAVES	2023	10.3758/s13428-023-02270-7	Normed affective stimulus database	Multimodal	discrete emotions	forced-choice identification	none
3TT	2023	10.1038/s41597-023-02015-3	Emotion-recognition dataset	Text	behavioural responses (similarity), valence	reaction times	none
ChillsDB	2023	10.1038/s41597-023-02064-8	Dynamic/multimodal stimulus set	Video	chills intensity, valence, arousal	5-point Likert	none
AKTIVES	2023	10.1038/s41597-023-02272-2	Psychophysiological response dataset	Physiological data	self-report stress, expert labels	expert labels	BVP, EDA, ST, facial coding
ReCANVo	2023	10.1038/s41597-023-02405-7	Domain-specific stimulus set	Audio	discrete emotions	not reported	none

					(affective function)		
not specified	2023	10.1038/s41598-023-33656-4	Dynamic/multimodal stimulus set	Video	discrete emotions (5), beauty, intensity	forced-choice, slider	none
RIKEN	2023	10.1038/s41598-023-49209-8	Dynamic/multimodal stimulus set	Faces	valence, arousal, discrete emotions, appraisals	SAM, free description	facial coding, depth
LitEmo	2023	10.1007/s42761-023-00219-9	Emotion-recognition dataset	Text	word frequencies, embeddings	not reported	none
Blu Pantheon dataset	2023	10.1049/ccs2.12076	Emotion-recognition dataset	Speech	discrete emotions	discrete emotion ratings	none
MuSe-CaR	2023	10.1109/taffc.2021.3097002	Emotion-recognition dataset	Multimodal	valence, arousal, trustworthiness, intensity, self-report ratings	dimensional ratings, Likert, discrete emotion ratings	none
eHRI	2023	10.1007/s10579-022-09632-1	Emotion-recognition dataset	Multimodal	none	not reported	none
Mementos	2023	10.1109/taffc.2021.3089584	Emotion-recognition dataset	Video	valence	dimensional ratings	none
EGEFILM	2023	10.3758/s13428-022-02055-4	Normed affective stimulus database	Video	valence, arousal, discrete emotions	Likert	none

not specified	2023	10.1017/s0272263123000463	Normed affective stimulus database	Speech	19 emotions, appraisals	5-point scales	none
DevEmo	2023	10.3390/app13063839	Emotion-recognition dataset	Video; Faces	discrete emotions	behavioural responses	FACS
UIBVFED-Mask	2023	10.3390/data8010017	Emotion-recognition dataset	Faces; Images	discrete emotions	not reported	none
Facial Expression Emotions	2023	10.3390/electronics12051089	Normed affective stimulus database	Faces; Images	discrete emotions	not reported	none
SEEC and CHASE	2023	10.1016/j.knosys.2023.111039	Emotion-recognition dataset	Text	discrete emotions	Likert	none
CEAP-360VR	2023	10.1109/tmm.2021.3124080	Psychophysiological response dataset	VR/AR; Physiological data; Eye-tracking	valence, arousal, discrete emotions	SAM; continuous ratings	BVP; EDA; SKT; HR; IBI; Eye-tracking
Werewolf-XL	2023	10.1109/taffc.2021.3101563	Dynamic/multimodal stimulus set	Faces; Audio; Speech; Video	18 non-prototypical emotions; PAD	discrete emotion ratings; PAD (SAM)	none
Life Events List	2023	10.1038/s41597-023-02483-7	Domain-specific stimulus set	Text	valence, significance	forced choice	none
ChildPredictor:	2023	10.1109/tmm.2022.3164785	Normed affective stimulus database	Faces	8 expresiones, intensidad, estimación de edad	porcentaje de acuerdo	none

REED	2023	10.1007/s10579-023-09698-5	Dynamic/multimodal stimulus set	Video	6 emociones básicas + neutral	precisión de clasificación	none
ViTaFa	2023	10.3758/s13428-023-02264-5	Dynamic/multimodal stimulus set	Multimodal	atractivo, arousal, claridad, genuinidad, familiaridad, intensidad, valencia	Likert 1-7	none
FENP	2023	10.1109/taffc.2020.3030296	Psychophysiological response dataset	Multimodal	pain-related emotions	labels by observers	Face video, sEMG, MoCap
AFDI	2023	10.1007/s41809-023-00130-6	Normed affective stimulus database	Video	valence, arousal, dominance, liking, familiarity	SAM	none
not specified	2023	10.1038/s41598-023-36346-3	Normative adaptation/translation	VR/AR	valence, arousal	SAM	EDA/GSR
OVPD	2022	10.1016/j.jneumeth.2022.109624	Psychophysiological response dataset	Multimodal; Video; Physiological data; Other (Odors)	valence; intensity	dimensional ratings; Likert	EEG
PDED	2022	10.11591/ijeecs.v26.i2.pp743-753	Psychophysiological response dataset	Physiological data	fear magnitude	discrete emotion ratings; Fscale	EEG
Multipic	2022	10.1038/s41597-022-01552-7	Normative adaptation/translation	Images	familiarity, naming agreement	Likert (0-100)	none

POPANE	2022	10.1038/s41597-021-01117-0	Psychophysiological response dataset	Physiological data; Multimodal	valence, discrete emotions, motivation	dimensional ratings; electronic scale	ECG; ICG; EDA; SBP/DBP; TEMP
MMEW	2022	10.1109/taffc.2022.3205170	Emotion-recognition dataset	Faces; Video	discrete emotions	FACS/AUs	none
not specified	2022	10.3758/s13428-021-01787-z	Normed affective stimulus database	Words	typicality, production frequency, mean response time, age of acquisition, concreteness	Likert, RT	none
not specified	2022	10.1109/access.2022.3146729	Psychophysiological response dataset	Multimodal	discrete emotions (6 basic + neutral)	discrete emotion ratings	EEG, EMG
WASABI Song Corpus v2	2022	10.1007/s10579-022-09601-8	Normed affective stimulus database	Multimodal	valence, arousal, topics, basic emotions	dimensional ratings, discrete ratings	none
MEVIEW	2022	10.1016/j.neucom.2022.06.101	Emotion-recognition dataset	Video	positive, negative, surprise	discrete emotion ratings	facial coding/AUs
RFAU	2022	10.1109/taffc.2020.3006392	Emotion-recognition dataset	Video	12 AUs and their intensities	six-point ordinal scale	FACS

AffectNet	2022	10.3390/s22218089	Emotion-recognition dataset	Images, Faces	valence, arousal, categorical emotions	dimensional, discrete	none
Chung-Ang	2022	10.1109/access.2022.3221453	Normed affective stimulus database	Audio, Speech	discrete emotions, naturalness	discrete ratings, Likert	none
not specified	2022	10.1109/taffc.2020.2978069	Psychophysiological response dataset	Physiological data	reflective thinking labels	behavioural responses	none (sensors)
KD-EmoR	2022	10.1109/access.2022.3221408	Domain-specific stimulus set	Text	euphoria, dysphoria, neutral	discrete emotion model	none
FEC	2022	10.1109/taffc.2019.2954498	ICT-3DRFE	Normed affective stimulus database	Faces (3D)	6 prototypic emotions, neutral	action unit classification
EDPE dataset	2022	10.32604/iasc.2022.020849	Psychophysiological response dataset	Physiological data	arousal, valence	dimensional ratings (-2 to +2)	PPG, EMG
Urdu multi-label emotion dataset	2022	10.7717/peerj-cs.896	Emotion-recognition dataset	Text	discrete emotions	expert annotations	none
Bi-AGI database	2022	10.3389/fpsyg.2022.948142	Normed affective stimulus database	Faces	trustworthiness, dominance, attractiveness	7-point Likert	none
not specified	2022	10.3758/s13414-022-02488-1	Normed affective stimulus database	Multimodal	self-report ratings	Not reported	none
IEFDB	2022	10.3758/s13428-022-01812-9	Normed affective stimulus database	Faces	discrete emotions, intensity	Select-all-that-apply	none

PLAViMoP	2022	10.3758/s13428-022-01850-3	Dynamic/multimodal stimulus set	Other (3D PLD)	self-report ratings	Not reported	none
Mandarin Chinese auditory emotion stimulus database	2022	10.3758/s13428-022-01868-7	Domain-specific stimulus set	Audio	discrete emotions, intensity	reconocimiento forzado	none
CLT	2022	10.3758/s13428-022-01923-3	Normed affective stimulus database	Images	valence, arousal, familiarity, intensity	Not reported	behavioural responses
SCOPE	2022	10.3758/s13428-022-01934-0	Normative adaptation/translation	Words	valence, arousal, dominance, self-report ratings	múltiples (SAM, etc.)	behavioural responses
CROWD-5e	2022	10.3758/s13428-022-02003-2	Normed affective stimulus database	Words	valence, arousal, discrete emotions, intensity	Likert	none
Authors' dataset	2022	10.3390/s22208066	Normed affective stimulus database	Faces	discrete emotions	discrete emotion ratings	none
MERP	2022	10.3390/s23010382	Dynamic/multimodal stimulus set	Audio	valence, arousal, self-report ratings	dimensional ratings	none
CNN aleatorios	2022	10.1016/j.engappai.2022.105349	Psychophysiological response dataset	Multimodal	valence, arousal, dominance, liking, familiarity	SAM	EEG, physiological data
not specified	2022	10.1109/tcds.2021.3055524	Domain-specific stimulus set	Video	discrete emotions	Likert	none

OBNIS	2022	10.1098/rsos.211128	Normative adaptation/translation	Images	valence, arousal, 12 new cognitive/affective dimensions	Likert (1 a 9)	none
VREED	2022	10.1016/j.bspc.2021.103349	Normed affective stimulus database	Audio	valence, arousal, discrete emotions	VAS, Likert	none
Urdu speech corpus	2022	10.7717/peerj-cs.954	Normed affective stimulus database	Speech	discrete emotions; self-report ratings	discrete emotion ratings	none
Not specified	2022	10.1177/02537176221111578	Normed affective stimulus database	Faces; Images	discrete emotions; intensity; clarity; genuineness	Likert	none
Misinformation Dataset	2022	10.1145/3477495.3531726	Psychophysiological response dataset	Physiological data; Text	credibility; familiarity; arousal	self-report ratings	EDA/GSR; PPG
DriverMVT	2022	10.3390/data7050062	Psychophysiological response dataset	Images; Physiological data	physiological responses; behavioural responses	physiological indicators	eye-tracking; EMG; skin temperature
BOOKAR	2022	10.1016/j.bspc.2022.103942	Psychophysiological response dataset	VR/AR; Text; Images	valence, arousal, dominance	SAM	EEG (Emotiv EPOC+)
SEMOUR+	2022	10.1007/s10579-022-09610-7	Emotion-recognition dataset	Speech	discrete emotions; valence,	discrete emotion ratings; PAD	none

					activation, dominance		
IDEST	2022	10.1371/journal.pone.0274480	Normed affective stimulus database	Text	valence, arousal, dominance, origin, significance, source	SAM	none
not specified	2022	10.1016/j.cogsys.2022.04.001	Dynamic/multimodal stimulus set	Faces	33 expresiones faciales y emociones	dificultad en la expresión	none
SUDFace	2022	10.3758/s13428-022-01951-z	Emotion-recognition dataset	Faces	expresiones faciales, iluminación	not reported	none
CFD Templates	2022	10.3758/s13428-022-01830-7	Normed affective stimulus database	Faces	edad, atractivo, babyfacedness, confiabilidad, inusual	Likert 1-7, Afrocentricidad 0-100	none
EmCat-Pol	2022	10.1515/psicl-2022-0028	Domain-specific stimulus set	Words	basic emotion synonyms	corpus/dictionary	none
EmCat-Eng	2022	10.2478/stap-2022-0003	Domain-specific stimulus set	Words	basic emotion synonyms	dictionary	none
VitaSi	2021	10.1016/j.compeleceng.2021.107392	Psychophysiological response dataset	Video; Physiological data	physiological responses; self-report ratings	not reported	ECG; respiration
FIP-affect	2021	10.1016/j.neucom.2021.01.016	Emotion-recognition dataset	Images	valence, arousal	SAM	none

Not explicitly named	2021	10.1109/jbhi.2020.3019242	Domain-specific stimulus set	Faces; Video	Clinical scores	facial landmark annotation	none reported (markers)
AVIATE	2021	10.1016/j.knosys.2021.107152	Unclear	Multimodal	Unclear in snip	Unclear	Unclear
MUMBAI	2021	10.1007/s12193-021-00364-0	Psychophysiological response dataset	Multimodal	affect (segment-level), experience, personality	segment-level annotations	behavioral (AUs)
MIST database	2021	10.3758/s13428-021-01639-w	Normed affective stimulus database	Words	valence, arousal, familiarity	SAM, Likert	none
AMIGOS	2021	10.1109/taffc.2018.2884461	Psychophysiological response dataset	Video, Physiological data	valence, arousal, dominance, liking	SAM, PANAS	EEG, ECG, EDA/GSR
BIOMEX-DB	2021	10.1109/access.2021.3100035	Psychophysiological response dataset	Multimodal, Speech	none reported	not reported	EEG
RECCON	2021	10.1007/s12559-021-09925-7	Domain-specific stimulus set	Text	discrete emotions, causes	discrete emotion model	none
DEAR-MULSEMEDIA	2021	10.1016/j.inffus.2020.08.007	Psychophysiological response dataset	Multimodal, Physiological data	valence, arousal, appreciation	SAM	EEG, EDA, PPG
LOCO	2021	10.3758/s13428-021-01698-z	Normed affective stimulus database	Text	valence, arousal, dominance (derived)	linguistic indicators	none
not specified	2021	10.1142/s0219467822500498	Normed affective stimulus database	Faces	discrete emotions, intensity	Likert	FACS
SOBEM	2021	10.3758/s13428-021-01640-3	Normed affective stimulus database	Faces	discrete emotions,	Not reported	none

					intensity, self-report ratings		
LinguaPix	2021	10.3758/s13428-021-01651-0	Normed affective stimulus database	Images	valence, arousal, familiarity, intensity	Likert	behavioural responses
Emotion Meanings	2021	10.3758/s13428-021-01697-0	Normed affective stimulus database	Words	valence, arousal, discrete emotions, self-report ratings	Likert	none
SpeakingFaces	2021	10.3390/s21103465	Psychophysiological response dataset	Multimodal	audio, skeletal landmarks	not reported	radar, laser, audio
SEMAINE	2021	10.1109/tpami.2019.2944808	Emotion-recognition dataset	Multimodal	valence, arousal, dominance, discrete emotions	dimensional ratings	none
MATTER	2021	10.3758/s13428-021-01567-9	Normed affective stimulus database	Images	valence, arousal	Likert (-2 a 2)	none
e-NatPOEM	2021	10.1038/s41598-021-91013-9	Domain-specific stimulus set	Images	disgust, fear, valence, arousal	Likert (1 a 9)	none
USID	2021	10.1007/s11292-021-09490-x	Domain-specific stimulus set	Images	threat, crime, neutral	not reported	none
MMEW	2021	10.1109/tpami.2021.3067464	Emotion-recognition dataset	Multimodal	valence, arousal, power	binary levels	none
DDPM	2021	10.1109/ijcb52358.2021.9484409	Emotion-recognition dataset	Video; Faces; Physiological data	credibility; physiological responses	behavioural responses	ECG/HR/HRV; blood oxygenation; Thermal

CORTEX	2021	10.3390/app112110146	Normative adaptation/translation	Text	self-report ratings	not reported	none
Shabd	2021	10.3758/s13428-021-01625-2	Domain-specific stimulus set	Words	valence, social desirability	Likert	none
CASIA-Face-Africa	2021	10.1109/tifs.2021.3080496	Emotion-recognition dataset	Faces	5 expresiones	not reported	none
RS-ANEW	2021	10.3758/s13428-021-01682-7	Normative adaptation/translation	Words	valence, arousal, imageability	SAM	none
SenseEmotion	2021	10.1109/taffc.2019.2892090	Psychophysiological response dataset	Multimodal	valence, arousal, dominance, pain	SAM	ECG, EDA, EMG, resp
DEAP Hybrid Model Tags	2020	10.1109/asyu50717.2020.9259797	Normative adaptation/translation	Audio; Video	discrete emotions; valence; arousal	dimensional ratings; discrete categories	none
e3learning	2020	10.1109/mmul.2019.2945716	Emotion-recognition dataset	Multimodal	discrete emotions	behavioural responses	none reported
not specified	2020	10.1109/taffc.2017.2768030	Psychophysiological response dataset	Physiological data; Video	valence	dimensional ratings	HR-EEG; GSR; ECG; respiration
FoCuS	2020	10.1002/mpr.1833	Domain-specific stimulus set	Multimodal; Video; Images	intensity, gaze accuracy	4AFC task; Likert	none
MEmoFC	2020	10.1007/s10579-020-09508-2	Domain-specific stimulus set	Text	affective language; discrete emotions	textual analysis (EmoMap)	none

CAAV	2020	10.1038/s41597-020-0366-1	Normed affective stimulus database	Video	valence, arousal	SAM, 9-point scale	none
FaReT Dataset	2020	10.3758/s13428-020-01421-4	Domain-specific stimulus set	Faces	trustworthiness, attractiveness, emotions	Likert, continuous	none
IESC-Child	2020	10.1016/j.csl.2019.06.006	Psychophysiological response dataset	Physiological data	valence, arousal	SAM	EEG
Delaware Pain Database (DPD)	2020	10.1097/pr9.0000000000000853	Domain-specific stimulus set	Faces	painfulness, discrete emotions, believability	Likert	FACS
UIBVFED	2020	10.1371/journal.pone.0231266	Emotion-recognition dataset	Faces, Images	discrete emotions, intensity	discrete emotion ratings	none
ERUFER	2020	10.1007/s11042-020-09268-9	Emotion-recognition dataset	Faces	discrete emotions	not reported	none
uulmMAC	2020	10.3390/s20082308	Psychophysiological response dataset	Multimodal, Video	clinical depression symptoms	BDI-II, self-report	respiration, facial coding
Tufts Face Database	2020	10.1109/tpami.2018.2884458	Emotion-recognition dataset	Images, Faces	facial expressions	not reported	none
K-EmoCon	2020	10.1038/s41597-020-00630-y	Psychophysiological response dataset	Multimodal, Video, Audio	arousal, valence, 18 emotions	dimensional, discrete, BROMP	EEG, ECG, EDA, temp

not specified	2020	10.1016/j.cmpb.2020.105571	Psychophysiological response dataset	Physiological data	discrete emotions	discrete emotion model, Likert	ECG, EDA/GSR
STUDENT ACTION	2020	10.1007/s11042-020-09741-5	Emotion-recognition dataset	Images	action labels	behavioural responses	none
SANDchild	2020	10.3758/s13428-020-01377-5	Normed affective stimulus database	Words	valence, arousal	SAM	none
Not specified	2020	10.1155/2020/8303465	(EEG Signals Database)	Psychophysiological response dataset	Physiological data; Video	discrete emotions	discrete emotion ratings
Not specified	2020	10.1109/tmm.2019.2922129	Emotion6 Video (Emotion6V)	Emotion-recognition dataset	Video	ekman-6 re-annotations, segments	discrete emotion ratings
not specified	2020	10.1371/journal.pone.0227754	Normed affective stimulus database	Images	aesthetic ratings	not reported	none
MedFaces	2020	10.1002/cae.22218	Psychophysiological response dataset	Multimodal	valence, arousal, personality	SAM	EEG, ECG, PI
Not specified	2020	10.1177/0301006620901671	Normed affective stimulus database	Video	valence, arousal, impact	SAM	none
GAPED	2020	10.3389/fpsyg.2020.02187	Normed affective stimulus database	Images	valence, arousal, moral/legal norms congruence	Escala 0-100	none
CEED	2020	10.1371/journal.pone.0228248	Domain-specific stimulus set	Video	valence (incidental)	agree-based classification	fMRI; MEG; EEG (intended)

not specified	2020	10.1007/s00138-020-01080-7	Normative adaptation/translation	Faces	precisión, tiempos de respuesta (RT), intensidad	calificación 7 expresiones	none
LSD	2020	10.3390/electronics9040661	Normed affective database	Images	7 dimensions (appeal, valence, etc.)	Likert	none
Tsinghua-FED	2020	10.1371/journal.pone.0231304	Normed affective stimulus database	Faces	8 emotions, intensity, age purity	choice list, indicators	none
DEVO	2020	10.1525/collabra.180	Normed affective stimulus database	Video	valence, arousal, impact	SAM	none
not specified	2020	10.1080/00224545.2020.1758016	Normed affective stimulus database	Video	valence, arousal, discrete emotions	Likert	none
SoPID	2020	10.3389/fpsyt.2020.00123	Dynamic/multimodal stimulus set	Video	discrete emotions	not reported	none
VAMPIR	2020	10.3758/s13428-019-01294-2	Psychophysiological response dataset	Words	valence, arousal	dimensional	EEG
Japanese vocalization set	2020	10.1037/emo0000580	Normed affective stimulus database	Speech	discrete emotions, intensity, valence, arousal	Categorization	none
VENEC Corpus	2020	10.1007/978-3-476-05728-0_14096-1	Normed affective stimulus database	Speech	19 emotions, appraisals	5-point scales	none
Famous Melodies Set	2020	10.3758/s13428-020-01411-6	Normed affective stimulus database	Audio	valence, arousal, familiarity	Likert	none

food-pics_extended	2019	10.3389/fpsyg.2019.00307	Normed affective stimulus database	Images	valence, arousal, palatability, familiarity	dimensional ratings; Likert	none reported
MHHRI	2019	10.1109/taffc.2017.2737019	Psychophysiological response dataset	Multimodal	personality, engagement, physiological responses	Likert	EEG, ECG, EDA, skin temperature
AM-FED+	2019	10.1109/taffc.2018.2801311	Emotion-recognition dataset	Video, Facial expression data	liking, familiarity	self-report ratings	FACS
DEMoS	2019	10.1007/s10579-019-09450-y	Normed affective stimulus database	Audio, Speech	valence, arousal, discrete emotions	dimensional, discrete ratings	none
EMOTIC	2019	10.1109/tpami.2019.2916866	Normed affective stimulus database	Images	behavioural traits inferences	Likert	none
not specified	2019	10.1007/s12144-018-0119-x	ATAL / ETAL	Normative adaptation/translation	Words	valence, arousal	SAM
LIRIS-CSE	2019	10.1016/j.imavis.2019.02.004	Dynamic/multimodal stimulus set	Video	discrete emotions	not reported	none
AffectNet	2019	10.1109/taffc.2017.2740923	Normed affective stimulus database	Images	valence, arousal, discrete emotions	dimensional ratings	none
not specified	2019	10.1002/ijop.12602	Normed affective stimulus database	Faces	discrete emotions	not reported	none
E-MOVIE	2019	10.1371/journal.pone.0223124	Normed affective stimulus database	Video	valence, arousal, discrete	SAM, Likert	none

					emotions, intensity		
Not specified	2019	10.3389/fpsyg.2019.00058	Normed affective stimulus database	Images	valence, arousal, desire-to-eat, perceived healthiness, familiarity	VAS	none
BAPS-Adult	2019	10.1016/j.psychres.2018.11.005	Domain-specific stimulus set	Images	valence, arousal, dominance, discrete emotions (distress, comfort, joy)	SAM, Likert	none
Spanish S-ANEW	2019	10.3389/fpsyg.2019.01988	Normed affective stimulus database	Words	valence, arousal, dominance	SAM	none
DEFSS	2019	10.1177/1747021819829012	Normed affective stimulus database	Faces	5 expresiones, identificación de intensidad	selección de lista, escala 1-7	estandarizado para EEG
MPED	2019	10.1109/access.2019.2891579	Psychophysiological response dataset	Physiological data	discrete emotions	Likert, dimensional	EEG, GSR, Resp, ECG
OL-SFED	2019	10.1049/iet-cvi.2018.5281	Emotion-recognition dataset	Multimodal	5 academic emotions	self/external annotation	none
MMI Face Database	2019	10.1007/978-3-662-54999-5_4	Dynamic/multimodal stimulus set	Multimodal	emotions, Action Units	FACS temporal segments	FACS
Baby Faces	2019	10.1016/j.jneumeth.2018.10.021	Normed affective stimulus database	Faces	discrete emotions	not reported	none

Yonsei Face DB	2019	10.3389/fpsyg.2019.02626	Normed affective stimulus database	Faces	valence, arousal, discrete emotions, intensity	Likert	none
Spontaneous MMI	2019	10.4324/9781315849454-3	Psychophysiological response dataset	Multimodal	discrete emotions	self-report ratings	FACS
not specified	2019	10.3758/s13428-019-01327-w	Normed affective stimulus database	Video	valence, arousal, intensity, memorability	dimensional ratings	none
ACASS	2019	10.3389/frobt.2019.00094	Domain-specific stimulus set	Video	discrete emotions	Likert	none
ANPW_R	2019	10.1177/1747021819834226	Normed affective stimulus database	Words	valence, arousal, origin, AoA	SAM	none
Ambiguity DB	2019	10.1017/s014271641900050x	Domain-specific stimulus set	Words	meaning-valence, NOM	Likert	none
Dutch Adjectives	2019	10.3758/s13428-019-01303-4	Normed affective stimulus database	Words	valence, arousal, AoA	Likert	none
ALoT (Duplicate)	2019	10.1007/s10936-019-09649-8	Domain-specific stimulus set	Words	valence, desirability	Likert	none
Animal.ID	2019	10.3390/ani9080475	Normed affective stimulus database	Images	valence, arousal, cuteness, familiarity, edibility, capacity to feel/think, kill acceptability, feelings of care	Likert	none

EmoMadrid	2019	10.1007/s11031-019-09780-y	Normed affective stimulus database	Images	valence, arousal	Likert (-2 a 2)	none
Food-Cal	2019	10.1007/s40519-019-00687-8	Domain-specific stimulus set	Images	valence, arousal, attractiveness, palatability	Likert	none
Objects-on-Hands	2019	10.1371/journal.pone.0219615	Domain-specific stimulus set	Images	valence, arousal, disgust, familiarity, name agreement	Likert	none
COMPASS	2019	10.1525/collabra.256	Normed affective stimulus database	Images	valence, arousal	Likert (1 a 9)	none
Wake Forest Alcohol Imagery Set	2019	10.1111/acer.14214	Domain-specific stimulus set	Images	valence, arousal, relation to alcohol	Likert/Slider (0-100)	none
MESS	2019	10.1044/2019_jslhr-s-19-0144	Normed affective stimulus database	Speech	activation, pleasantness, discrete emotions	SAM, sliders	none
HVEC	2019	10.3758/s13428-019-01288-0	Normed affective stimulus database	Speech	discrete emotions	forced choice	none
JESS	2019	10.3758/s13428-019-01296-0	Normed affective stimulus database	Speech	attractiveness, likeability, age	Likert	none
not specified	2019	10.3389/fpsyg.2019.00757	Dynamic/multimodal stimulus set	Faces, Video	discrete emotions, intensity	categorical emotion ratings, Likert	none reported
MEMOA	2019	10.1109/acii.2019.8925462	Psychophysiological response dataset	Multimodal	valence, arousal	dimensional ratings	ECG, HR, movement

SMID	2018	10.1371/journal.pone.0190954	Normed affective stimulus database	Images	valence, arousal, moral wrongness, moral values	Likert	none
C-DIS	2018	10.3389/fpsyg.2018.01397	Domain-specific stimulus set	Images	disgust intensity, valence	Likert-type ratings	none
Natural Disasters Picture System (NDPS)	2018	10.1371/journal.pone.0201942	Domain-specific stimulus set	Images	valence, arousal, dominance, certainty	SAM	none
CAS(ME)2	2018	10.1109/taffc.2017.2654440	Emotion-recognition dataset	Faces, Video	discrete emotions, arousal intensity	discrete emotion model	FACS
not specified	2018	10.3389/fpsyg.2018.02358	(East Asian Face Emotion Stimuli)	Normed affective stimulus database	Faces	6 basic emotions, intensity, valence	discrete ratings, Valence (-4 a 4)
FIDENTIS 3D Face Database	2018	10.2478/anre-2018-0016	Domain-specific stimulus set	Faces	none (morphology)	not reported	none
Indonesian Mobile Video Dataset	2018	10.11591/ijece.v8i5.pp4042-4046	Emotion-recognition dataset	Multimodal	authentic emotions	JSON annotations	none
CaNAFF	2018	10.5334/irsp.179	Normed affective stimulus database	Faces	emotional neutrality, attractiveness, prototypicality, approach/avoidance tendencies	dimensional ratings	none

Dynamic FACES	2018	10.1080/02699931.2018.1445981	Normed affective stimulus database	Faces	discrete emotions, perceived age	not reported	none
EXCEED	2018	10.1371/journal.pone.0204093	Domain-specific stimulus set	Images	valence, arousal	SAM, Likert	none
GBPS	2018	10.1016/j.drugalcdep.2017.11.022	Domain-specific stimulus set	Images	valence, arousal, control, urge to drink, familiarity	not reported	none
IADS-E	2018	10.3758/s13428-018-1027-6	Normed affective stimulus database	Audio	valence, arousal, dominance, discrete emotions	SAM, 9-point Likert	none
SAMM	2018	10.1109/taffc.2016.2573832	Dynamic/multimodal stimulus set	Video	valencia, dominancia, arousal, emociones discretas	SAM (9 puntos), intensidad 0-4	FACS
CAFE subset	2018	10.1371/journal.pone.0209644	Normative adaptation/translation	Faces	familiaridad, atractivo, arousal, in-group, valencia, claridad, intensidad, genuinidad	8 dimensiones evaluativas subjetivas	none
DREAMER	2018	10.1109/jbhi.2017.2688239	Psychophysiological response dataset	Multimodal	valence, arousal, dominance	dimensional ratings	MEG, hEOG, ECG, tEMG, Face Video

SATED	2018	10.1371/journal.pone.0190921	Dynamic/multimodal stimulus set	Video	valence, arousal, naturalness	Likert, SAM	motion energy
KDEF-dyn	2018	10.1038/s41598-018-35259-w	Dynamic/multimodal stimulus set	Video	probability of fixation, entry time, duration	categorization, tracking metrics	eye-tracking
MUG database	2018	10.31234/osf.io/qfzsm	Psychophysiological response dataset	Multimodal	6 basic emotions (induced)	categorical labeled	facial landmarks
Sequential facial dataset	2018	10.1109/fg.2018.00098	Emotion-recognition dataset	Video	not specified	not reported	none
RaFD (Indian Norms)	2018	10.1371/journal.pone.0203959	Normative adaptation/translation	Faces	valence, arousal, discrete emotions, intensity	Likert	none
Brazilian teenagers face set	2018	10.1177/0301006618797226	Normed affective stimulus database	Faces	discrete emotions	forced-choice	none
KDEF-dyn	2018	10.3389/fpsyg.2018.02052	Dynamic/multimodal stimulus set	Video	discrete emotions, AUs	human categorization	FACS
not specified	2018	10.1080/02699931.2018.1530197	Normed affective stimulus database	Video	valence, arousal, discrete emotions, intensity	Likert	none
MAAFS	2018	10.1371/journal.pone.0206604	Domain-specific stimulus set	Video	discrete emotions, intensity	Likert	none
not specified	2018	10.1109/taffc.2017.2660485	Psychophysiological response dataset	Multimodal	valence, arousal, dominance, discrete	SAM, PANAS	EEG

					emotions, liking, familiarity		
WADAMO	2018	10.1177/0301006618816631	Domain-specific stimulus set	Video	liking, beauty, expressivity	Likert, slider	none
Bonin Norms	2018	10.3758/s13428-018-1014-y	Normed affective stimulus database	Words	concreteness, valence	Likert	none
Glasgow Norms	2018	10.3758/s13428-018-1099-3	Normed affective stimulus database	Words	arousal, valence, AoA	Likert	RTs
Best-Worst Norms	2018	10.3758/s13428-017-1009-0	Normed affective stimulus database	Words	valence, AoA	best-worst	none
EU-Emotion Voice Database	2018	10.3758/s13428-018-1048-1	Normed affective stimulus database	Speech	valence, intensity, arousal	5-point Likert	none
RAVDESS	2018	10.1371/journal.pone.0196391	Dynamic/multimo dal stimulus set	Audio, Video	discrete emotions, intensity	categorical labels, Likert	none reported
MSP-IMPROV	2017	10.1109/taffc.2016.2515617	Dynamic/multimo dal stimulus set	Multimodal; Video; Audio	valence, activation, dominance, emotions	dimensional ratings; SAM; Likert	none
BAUM-1	2017	10.1109/taffc.2016.2553038	Emotion-recognition dataset	Multimodal	discrete emotions, intensity	discrete emotion ratings	none
COGNIMUSE	2017	10.1186/s13640-017-0194-1	Emotion-recognition dataset	Multimodal	valence, arousal	dimensional ratings	none
not specified	2017	10.1080/00221325.2017.135141 6	Normed affective stimulus database	Images	valence, arousal, dominance, discrete	SAM	none

					emotions, intensity		
MONS	2017	10.3389/fpsyg.2017.01669	Domain-specific stimulus set	Images	valence, arousal, motivation (approach/avoid, interaction), recognizability	Likert (1 a 7)	none
PiSCES	2017	10.3758/s13428-017-0947-x	Normed affective stimulus database	Images	valence, intensity, social engagement	Likert (1 a 7)	none
CASMEMG	2017	10.1007/s00048-017-0180-9	Emotion-recognition dataset	Faces; Physiological data	discrete emotions	discrete emotion ratings	EMG
OxVoc Extension	2017	10.1037/pas0000382	Normed affective stimulus database	Audio	valence	VAS	none
AFEW-VA	2017	10.1016/j.imavis.2017.02.001	Not applicable	Words	valence	not reported	none
not specified	2017	10.1007/s10919-017-0268-x	Normative adaptation/translation	Faces	genuinidad percibida, precisión de etiquetado, intensidad	escala punto medio neutral, 1-9	none
TIF	2017	10.3389/fpsyg.2017.00409	Normed affective stimulus database	Faces	expresión, intensidad, claridad, genuinidad, valencia	escalas de 5 puntos	none

not specified	2017	10.1109/tmm.2017.2699859	Dynamic/multimodal stimulus set	Video, Text	discrete emotions, intensity, valence	categorical, Likert	none
DuckEES	2017	10.1002/mpr.1553	Dynamic/multimodal stimulus set	Video	7 emotions	semi-forced choice	none
GFT database	2017	10.1109/fg.2017.144	Psychophysiological response dataset	Video	FACS occurrence, intensity	binary presence/absence	FACS, landmarks
ISED	2017	10.1109/taffc.2015.2498174	Dynamic/multimodal stimulus set	Video	4 emotions, intensity	self-report, expert	none
City Infant Faces DB	2017	10.3758/s13428-017-0859-9	Normed affective stimulus database	Faces	intensity, clarity, genuineness	5-point scales	none
DaFEx	2017	10.3389/fpsyg.2017.00855	Dynamic/multimodal stimulus set	Video	6 emotions + neutral	classification accuracy	none
CAFE child validation	2017	10.1080/02699931.2017.1365046	Normative adaptation/translation	Faces	identification accuracy	label selection	none
BNU-LSVED 2.0	2017	10.1016/j.image.2017.08.012	Psychophysiological response dataset	Video	PAD (valence, arousal, dominance), discrete emotions	Likert, SAM	none
IAPD	2017	10.1002/ijop.12471	Normed affective stimulus database	Faces	discrete emotions, intensity	Likert	none
not specified	2017	10.3758/s13428-016-0842-x	Normative adaptation/translation	Video	discrete emotions	Likert	none

not specified	2017	10.3389/fpsyg.2017.01941	Normed affective stimulus database	Video	valence, arousal, dominance, discrete emotions, familiarity, liking	SAM, Likert	ECG/HR/HRV, respiration
not specified	2017	10.3389/fpsyg.2017.02116	Domain-specific stimulus set	VR/AR	valence, arousal	SAM	motion capture
NAA	2017	10.1109/acii.2017.8273574	Psychophysiological response dataset	Multimodal	valence, arousal, dominance, aggression, fear	dimensional ratings	ECG, GSR, EMG, motion capture
not specified	2017	10.1109/acii.2017.8273582	Psychophysiological response dataset	Multimodal	valence, arousal	SAM	none
Dutch Modality Norms	2017	10.3758/s13428-017-0852-3	Domain-specific stimulus set	Words	sensory modality	0-5 sliders	none
DTN	2017	10.3758/s13428-017-0890-x	Domain-specific stimulus set	Words	valence, arousal, tabooness	SAM/Likert	none
Italian ANEW	2017	10.1371/journal.pone.0169472	Normative adaptation/translation	Words	valence, arousal, imageability	SAM	none
DIRTI	2017	10.1016/j.brat.2016.11.010	Domain-specific stimulus set	Images	disgust, fear, valence, arousal	Likert (1 a 9)	none
CaTIS	2017	10.1007/s11292-017-9314-2	Domain-specific stimulus set	Images	threat, crime, neutral ratings	not reported	none
BPD-relevant set	2017	10.1037/per0000173	Normative adaptation/translation	Images	arousal	SAM	physiological sensors

Irish Political Speech Database	2017	10.1007/s10579-017-9401-z	Domain-specific stimulus set	Speech	charisma, persuasiveness, boredom, etc.	Likert	none reported
not specified	2017	10.4324/9781315229386-13	Not applicable	Not applicable	none	not reported	none reported
EmoLiTe	2017	10.1109/aciiw.2017.8272587	Psychophysiological response dataset	Text, Eye-tracking	interest, dislike, emotions	SAM, PANAS	video, eye-tracking
EMOTHAW	2017	10.1109/thms.2016.2635441	Psychophysiological response dataset	Handwriting	anxiety, depression, stress	DASS-42	pen pressure, position
LEED	2017	10.3758/s13428-017-0878-6	Normed affective database	Images	valence, arousal, appeal, etc.	Likert	none reported
JESTKOD	2016	10.1007/s10579-016-9377-0	Dynamic/multimodal stimulus set	Multimodal; Audio; Video	activation, valence, dominance	dimensional ratings; continuous affect	motion capture
Music Video Dataset,	2016	10.1145/2926719	Music Video Dataset (MVD)	Emotion-recognition dataset	Video	mood tags (ground-truth)	discrete emotion ratings
EMMA	2016	10.1145/2897369	Emotion-recognition dataset	Multimodal	valence, arousal, discrete emotions	SAM	motion capture
OASIS	2016	10.3758/s13428-016-0715-3	Normed affective stimulus database	Images	valence, arousal	Likert (1 a 7)	none
The Minho Word Pool	2016	10.3758/s13428-016-0767-4	Normative adaptation/translation	Words	valence, arousal, dominance	SAM	none

EMOTE	2016	10.1177/0033294116658474	Normed affective stimulus database	Words	valence, arousal, emotionality, imagery, familiarity, control, desirability	Likert	behavioral responses
Extended Cohn-Kanade (CK+)	2016	10.3906/elk-1401-18	Psychophysiological response dataset	Video	7 emociones discretas, códigos FACS	criterios FACS, intensidad 1-7	FACS, landmarks
MuDERI	2016	10.1007/978-3-319-47437-3_26	Psychophysiological response dataset	Multimodal	discrete emotions, V/A	SAM, Likert	EEG, EDA, RGB-D video
perceived-as-genuine/fake sets	2016	10.3758/s13428-016-0813-2	Normative adaptation/translation	Faces	genuineness, accuracy, intensity	neutral-midpoint scale, 1-9 scale	none
DEFSS	2016	10.3758/s13428-016-0756-7	Normed affective stimulus database	Faces	5 expressions, intensity	choice list, 1-7 scale	none
SAVE database	2016	10.3758/s13428-016-0790-5	Dynamic/multimodal stimulus set	Multimodal	attractiveness, arousal, clarity, etc.	Likert 1-7	none
ADFES-BIV	2016	10.1371/journal.pone.0147112	Dynamic/multimodal stimulus set	Video	discrete emotions, intensity	forced-choice	none
BNU-LSVED	2016	10.1117/12.2235892	Dynamic/multimodal stimulus set	Video	learning emotions (discrete)	not reported	none
BIC-Multicolor	2016	10.1037/pne0000040	Normed affective stimulus database	Faces	friendliness, race categorization	Likert	none

Kinect Facial Protocol	2016	10.1109/wacv.2016.7477559	Not applicable	Facial expression data	none	not reported	FACS
DISFA+	2016	10.1109/cvprw.2016.182	Psychophysiological response dataset	Video	intensity of FACS actions, self-report	6-point scale	FACS
Aff-Wild	2016	10.1109/cvprw.2016.186	Emotion-recognition dataset	Video	valence, arousal	continuous ratings	FACS
CHEAVD	2016	10.1007/s12652-016-0406-z	Emotion-recognition dataset	Multimodal	discrete emotions	discrete emotion ratings	none
ANPST	2016	10.3389/fpsyg.2016.01030	Normed affective stimulus database	Text	valence, arousal, origin, significance	SAM	none
CEFI	2016	10.3389/fpsyg.2016.01907	Normed affective stimulus database	Words	valence, arousal, dominance	1-to-9 scale	none
FAN	2016	10.3758/s13428-016-0831-0	Normed affective stimulus database	Words	valence, arousal	SAM	none
Dyspnoea Word Set	2016	10.1016/j.resp.2015.12.006	Domain-specific stimulus set	Words	valence, arousal	VAS	none
Spanish Discrete	2016	10.3758/s13428-016-0768-3	Normative adaptation/translation	Words	happiness, anger, fear	Discrete ratings	none
not specified	2016	10.3758/s13428-015-0700-2	Normed affective stimulus database	Words	valence, arousal	Likert	none
Ambiguity Norms	2016	10.1017/s0142716416000266	Domain-specific stimulus set	Words	valence, arousal, NOM	Likert	none

kidBAWL	2016	10.3389/fpsyg.2016.00969	Normative adaptation/translation	Words	valence, arousal	SAM	none
DRM Word Lists	2016	10.1080/02699931.2016.1138930	Domain-specific stimulus set	Words	valence, arousal	not reported	none
not specified	2016	10.3758/s13428-016-0703-7	Normed affective stimulus database	Images	valence, body shape	Likert	none
OLAF	2016	10.1371/journal.pone.0158991	Domain-specific stimulus set	Images	valence, arousal, dominance, food craving	SAM	none
MAPS	2016	10.1016/j.jbtep.2015.07.006	Domain-specific stimulus set	Images	valence, arousal, dominance	SAM	none
SFIP	2016	10.3758/s13428-016-0797-y	Domain-specific stimulus set	Images	fear, arousal, valence	SAM, fear scale (1-5)	none
ToMenovela	2016	10.3389/fpsyg.2016.01883	Domain-specific stimulus set	Images	valence, discrete emotions, affective ToM, salience	VAS	none
Musical bank (U1-U120)	2016	10.1177/0305735616671587	Normed affective stimulus database	Audio	valence, arousal, dominance, imageability	SAM	none
Lepping Musical Set	2016	10.1177/0305735615604509	Normed affective stimulus database	Audio	valence, arousal	Biaxial clicking	none
BioVid Emo DB	2016	10.1109/ssci.2016.7849931	Psychophysiological response dataset	Multimodal	discrete emotions, intensity, valence, arousal	Likert, numerical ratings	EDA/GSR, ECG, EMG

not specified	2016	10.1080/00224545.2016.1208138	Dynamic/multimodal stimulus set	Video, Text	discrete emotions, intensity, valence	categorical, Likert	none reported
EmoPain	2016	10.1109/taffc.2015.2462830	Psychophysiological response dataset	Multimodal	pain-related emotions	labels by observers	mocap, sEMG
MAHNOB Mimicry	2015	10.1016/j.patrec.2015.03.005	Dynamic/multimodal stimulus set	Multimodal; Video; Audio	discrete emotions; mimicry	categorical assessment	none reported (markers)
BAES-DB	2015	10.3813/aaa.918899	Emotion-recognition dataset	Speech	discrete emotions (joy, anger, sadness)	discrete emotion ratings	none
NAWL	2015	10.3758/s13428-014-0552-1	Normative adaptation/translation	Words	valence, arousal, imageability	SAM	none
MADS	2015	10.3758/s13428-015-0572-5	Normed affective stimulus database	Video	valence, arousal, dominance, liking, familiarity	SAM	none
NOUN	2015	10.3758/s13428-015-0647-3	Normed affective stimulus database	Speech	attractiveness, likability, age	Likert	none
NAWL BE	2015	10.1371/journal.pone.0132305	Normed affective stimulus database	Words	discrete emotions (happiness, anger, sadness, fear, disgust)	discrete emotion ratings (intensity)	none
RaFD	2015	10.3389/fpsyg.2014.01516	Normed affective stimulus database	Faces	8 expresiones, intensidad, claridad,	elección forzada, escalas 5 puntos	FACS

					genuinidad, atractivo, valencia		
The MR2	2015	10.3758/s13428-015-0641-9	Normed affective stimulus database	Faces	edad, raza percibida, atractivo, humor	escalas Likert 1-7	none
CAM Face-Voice Battery	2015	10.1186/s13229-015-0018-z	Emotion-recognition dataset	Multimodal	complex emotions	4-label forced-choice	none
CAFE set	2015	10.3389/fpsyg.2014.01532	Normed affective stimulus database	Faces	6 emotions + neutral	forced-choice identification	none
CFD	2015	10.3758/s13428-014-0532-5	Normed affective stimulus database	Faces	age, trust, babyfacedness, etc.	Likert 1-7	none
NIMH-ChEFS (revised)	2015	10.1002/mpr.1490	Normative adaptation/translation	Faces	accuracy, representativeness	selection, 1-10 slider	none
UNCEEF	2015	10.1080/02699931.2015.1098590	Normed affective stimulus database	Faces	discrete emotions	forced-choice	FACS
not specified	2015	10.3758/s13428-015-0654-4	Dynamic/multimodal stimulus set	Multimodal	discrete emotions, intensity	discrete emotion ratings	none
not specified	2015	10.1080/02699931.2015.1031089	Normed affective stimulus database	Video	discrete emotions	Likert	none
LIRIS-ACCEDE	2015	10.1109/taffc.2015.2396531	Normed affective stimulus database	Video	valence, arousal	dimensional ratings	none
Moral Vignettes	2015	10.3758/s13428-014-0551-2	Domain-specific stimulus set	Text	moral ratings, valence	Likert	none

not specified	2015	10.3758/s13428-015-0684-y	Domain-specific stimulus set	Words (stems)	completion probability	task indices	none
Action Words	2015	10.7717/peerj.1100	Domain-specific stimulus set	Words	discrete associations	rating tasks	none
PANIG	2015	10.3758/s13428-015-0581-4	Domain-specific stimulus set	Words (idioms)	valence, figurativeness	Likert	none
BAPS-Ado	2015	10.1016/j.psychres.2015.04.05 5	Domain-specific stimulus set	Images	valence, arousal, dominance, discrete emotions	SAM, Likert	none
ABPS	2015	10.1111/acer.12853	Domain-specific stimulus set	Images	valence, arousal, control, urge to drink, familiarity, history	not reported	none
Italian pseudowords corpus	2015	10.3758/s13428-015-0570-7	Normed affective stimulus database	Speech	valence, arousal, discrete emotions	VAS	none
DECAF	2015	10.1109/taffc.2015.2392932	Psychophysiologic al response dataset	Multimodal	valence, arousal, dominance	dimensional ratings	MEG, hEOG, ECG, tEMG, Face Video
LSD	2015	10.3758/s13428-015-0643-7	Normed affective database	Images	appeal, familiarity, valence, etc.	Likert	none reported
NaLMC	2015	10.1145/2818346.2820772	Dynamic/multimo dal stimulus set	Audio, Video	discrete emotions	GEW, Ekman labels	none reported

CreativeIT	2015	10.1007/s10579-015-9300-0	Psychophysiological response dataset	Multimodal	activation, valence, dominance	Feeltrace, Likert	mocap, microphones
EU-Emotion Stimulus Set	2015	10.3758/s13428-015-0601-4	Dynamic/multimodal stimulus set	Multimodal	20 emotions, V/A	categorical, dimensional	none reported
UMEME	2015	10.1109/taffc.2015.2407898	Dynamic/multimodal stimulus set	Multimodal	discrete emotions, V/A/D	Likert, SAM	none reported
GEFAV	2014	10.3758/s13428-014-0545-0	Normed affective stimulus database	Multimodal	attractiveness, health, dominance, trustworthiness	continuous scale (0-100)	none
LIRIS	2014	10.1109/tsmc.2014.2331215	Normed affective stimulus database	Multimodal (2D/3D)	facial expressions	not reported	none
not specified	2014	10.3758/s13428-014-0494-7	(Sociality Word Database)	Domain-specific stimulus set	Words	valence, arousal, potency, authority, community	Semantic-differential
Not specified	2014	10.3758/s13428-014-0491-x	Dynamic/multimodal stimulus set	Video	discrete emotions	not reported	EEG, ERP
Food-pics	2014	10.3389/fpsyg.2014.00617	Domain-specific stimulus set	Images	valence, arousal, familiarity, palatability, complexity	Likert	none
OxVoc Sounds database	2014	10.3389/fpsyg.2014.00562	Normed affective stimulus database	Audio	valence, arousal, motivation	VAS, Likert	none

BP4D-Spontaneous	2014	10.1016/j.imavis.2014.06.002	Dynamic/multimodal stimulus set	Faces (3D dynamic)	discrete emotions; intensity; FACS	Likert (0-5)	FACS
ANGST	2014	10.3758/s13428-013-0426-y	Normed affective stimulus database	Text	valence, arousal, dominance	SAM	none
CREMA-D	2014	10.1109/taffc.2014.2336244	Dynamic/multimodal stimulus set	Multimodal	7 estados emocionales	experimentos de escucha	none
MPI EMBM	2014	10.1371/journal.pone.0113647	Dynamic/multimodal stimulus set	Video, Mocap	11 emotions, naturalness	categorical	motion capture
FEEDB	2014	10.1007/978-3-319-06932-6_37	Dynamic/multimodal stimulus set	Faces	33 facial expressions and emotions	difficulty in expressing	none
CASIA Natural Emotional	2014	10.1109/icosp.2014.7015071	Dynamic/multimodal stimulus set	Multimodal	discrete emotions	not reported	none
CASME II	2014	10.1371/journal.pone.0086041	Psychophysiological response dataset	Video	discrete emotions	self-report ratings	FACS
McEwan Faces	2014	10.1371/journal.pone.0088783	Normed affective stimulus database	Faces	kindness, criticism, neutrality	forced-choice	none
not specified	2014	10.3758/s13428-013-0439-6	Normed affective stimulus database	Video	discrete emotions, intensity	discrete emotion ratings	none
not specified	2014	10.1068/p7581	Domain-specific stimulus set	Video	valence, arousal, liking, familiarity, beauty, interest	Likert	none

BAUM-2	2014	10.1007/s11042-014-1986-2	Emotion-recognition dataset	Multimodal	discrete emotions, intensity	discrete emotion ratings	none
not specified	2014	10.1016/j.pain.2014.08.019	Domain-specific stimulus set	Video	valence, arousal	dimensional ratings	none
not specified	2014	10.3758/s13428-014-0500-0	Normative adaptation/translation	Video	valence, arousal, discrete emotions, intensity	SAM, Likert	none
Human Attributes	2014	10.1037/cep0000001	Normed affective stimulus database	Words	valence, intensity, familiarity	7-point Likert	none
ANPW	2014	10.3758/s13428-014-0509-4	Normed affective stimulus database	Words	valence, arousal, origin, source	SAM	none
Basic Emotion Ratings	2014	10.1109/tmm.2014.2357688	Normative adaptation/translation	Words	discrete emotion intensity	Likert	none
OLAF (Adolescents)	2014	10.1371/journal.pone.0114515	Normative adaptation/translation	Images	valence, arousal, dominance, food craving	SAM	none
EMOVO	2014	10.63317/4i4sxkp59t9f	Normed affective stimulus database	Speech	discrete emotions	Categorization	none
MIAS	2014	10.3758/s13428-014-0445-3	Normed affective stimulus database	Audio	valence, arousal, discrete emotions	VAS, Likert	none
Persian ESD	2014	10.3758/s13428-014-0467-x	Normative adaptation/translation	Speech	not specified	not reported	none reported

Emilya	2014	10.63317/3x4br8qxnyb9	Dynamic/multimodal stimulus set	Video, Mocap	8 emotions	perceived emotion matching	Motion capture
Inter-ACT	2013	10.1142/s0219843613500102	Emotion-recognition dataset	Multimodal	valence, level of interest, engagement	discrete level ratings	none
Dartmouth Children's Faces	2013	10.1371/journal.pone.0079131	Normed affective stimulus database	Faces	discrete emotions, intensity, perceived age	Likert-type intensity	none
MAHNOB Laughter	2013	10.1016/j.imavis.2012.08.014	Psychophysiological response dataset	Multimodal, Video, Audio	laughter, speech	behavioural responses	none (video/audio)
Corpus of nonverbal vocalizations	2013	10.3758/s13428-013-0324-3	Normed affective stimulus database	Speech	valence, arousal, authenticity	7-point scales	none
CMU PIE	2013	10.1109/tpami.2012.206	Emotion-recognition dataset	Faces	neutral, smile, blink, etc.	not reported	none
NAPS	2013	10.3758/s13428-013-0379-1	Normative adaptation/translation	Words, Audio	valence, arousal	dimensional ratings	none
PFA-U	2013	10.1108/s1746-9791(2013)0000009011	Normed affective stimulus database	Faces	6 emotions	forced-choice, recognition rates	FACS
AM-FED	2013	10.1109/cvprw.2013.130	Psychophysiological response dataset	Video	liking, familiarity	discrete emotion ratings	FACS

NVIE (Analysis)	2013	10.1109/t-affc.2012.32	Normative adaptation/translation	Multimodal	valence, arousal, discrete emotions, intensity	self-report ratings	none
BESST	2013	10.1016/j.psychres.2012.11.012	Normed affective stimulus database	Faces, Bodies	discrete emotions, naturalness	Likert	none
CASME	2013	10.1109/fg.2013.6553799	Psychophysiological response dataset	Video	discrete emotions	self-report ratings	FACS
Dynamic virtual faces set	2013	10.1007/s10055-013-0236-7	Dynamic/multimodal stimulus set	VR/AR	discrete emotions	PANAS	none
DISFA	2013	10.1109/t-affc.2013.4	Psychophysiological response dataset	Video	valence, AU intensity	6-point scale	FACS
DynEmo	2013	10.5121/ijma.2013.5505	Psychophysiological response dataset	Video	affective states, emotions	dimensional, continuous	none
Extended ChaeLee	2013	10.4306/pi.2013.10.2.155	Normed affective stimulus database	Faces	valence, arousal, discrete emotions	not reported	none
ISED / IMFDB	2013	10.1109/ncvpripg.2013.6776225	Dynamic/multimodal stimulus set	Video	discrete emotions	not reported	none
LIRIS-ACCEDE	2013	10.1109/acii.2013.9	Emotion-recognition dataset	Video	valence	dimensional ratings	none
CCDb	2013	10.1109/cvprw.2013.48	Emotion-recognition dataset	Video	none	not reported	none

Moral Dilemmas Set	2013	10.1002/bdm.1782	Domain-specific stimulus set	Text	valence, arousal, moral acceptability	Likert	none
Category norms	2013	10.3758/s13428-012-0314-x	Not applicable	Words	valence	not reported	none
Finnish Nouns	2013	10.1371/journal.pone.0072859	Normed affective stimulus database	Words	valence, arousal	dimensional	none
FRIDa	2013	10.3389/fnhum.2013.00051	Domain-specific stimulus set	Images	valence, arousal, familiarity, calorie content, typicality, ambiguity	0-100 scale	none
not specified	2013	10.1080/09658211.2013.770871	Not applicable	Audio	memory, identification	Forced choice	none
MEB	2013	10.3389/fpsyg.2013.00509	Normed affective stimulus database	Audio	valence, arousal, discrete emotions	forced-choice	none
RECOLA	2013	10.1109/fg.2013.6553805	Psychophysiological response dataset	Multimodal	arousal, valence, social primitives	dimensional ratings	ECG, EDA
MMLI	2013	10.1007/978-3-319-02714-2_16	Dynamic/multimodal stimulus set	Multimodal	laughter episodes	not reported	mocap, audio, video
French speech corpus	2012	10.1016/j.specom.2011.10.005	Emotion-recognition dataset	Speech	basic emotions, attitudes	discrete ratings	none
ICT-3DRFE	2012	10.1016/j.imavis.2012.02.001	Dynamic/multimodal stimulus set	Video (3D)	discrete emotions, facial actions	not reported	none

not specified	2012	10.1017/brimp.2012.18	Normed affective stimulus database	Video	discrete emotions, intensity	VAS	none
IBGHT	2012	10.1007/s11042-012-1212-z	Psychophysiological response dataset	Video	ocurrencia de FACS, intensidad	presencia/ausencia binaria	FACS, landmarks
LDOS-PerAff-1	2012	10.1007/s12193-012-0107-7	Normed affective stimulus database	Faces	6 emociones básicas + neutral	elección forzada	none
The MPI Facial Expression Database	2012	10.1371/journal.pone.0032321	Dynamic/multimodal stimulus set	Multimodal	55 expresiones emocionales y conversacionales	nombrado libre	3D scans, head-tracking
Umeå University Database of Facial Expressions	2012	10.2196/jmir.2196	Normed affective stimulus database	Faces	8 expresiones, proporción correcta	elección semi-forzada, intensidad 1-7	none
EMDB	2012	10.1007/s10484-012-9201-6	Emotion-recognition dataset	Text	valence, arousal, discrete emotions	dimensional ratings	none
BINED	2012	10.1109/t-affc.2011.26	Psychophysiological response dataset	Video	self-report, valence/intensity trace	FeelTrace, Likert	none
AFEW / SFEW	2012	10.1109/mmul.2012.26	Dynamic/multimodal stimulus set	Video	discrete emotions	not reported	none
not specified	2012	10.1115/1.859988.ch4	Dynamic/multimodal stimulus set	Multimodal	valence, arousal, discrete emotions	FEELTRACE	none
DEAP	2012	10.1109/t-affc.2011.15	Psychophysiological response dataset	Multimodal	valence, arousal, dominance,	SAM	EEG, physiological data

					liking, familiarity		
SAWL	2012	10.1017/s0142716412000409	Normed affective stimulus database	Words	valence, arousal, familiarity, imageability	Likert	fMRI
French Traits	2012	10.3758/s13428-012-0276-z	Domain-specific stimulus set	Words	valence, consequences	Likert	none
LANG	2012	10.1371/journal.pone.0030086	Normed affective stimulus database	Words	valence, arousal	SAM	fMRI/EEG
Mandarin vocal emotional database	2012	10.3758/s13428-012-0203-3	Normed affective stimulus database	Speech	discrete emotions, intensity	5-point Likert	none
MAHNOB-HCI	2012	10.1109/t-affc.2011.25	Psychophysiologic al response dataset	Multimodal	valence, arousal, dominance, predictability	SAM	EEG, ECG, EDA, respiration, eye-tracking
GEMEP-CS	2012	10.1037/a0025827	Dynamic/multimo dal stimulus set	Multimodal	15 emotions, intensity	categorical, Likert	facial coding
EmoTales	2011	10.1007/s10579-011-9140-5	Emotion-recognition dataset	Text	evaluation, activation, power, basic emotions	SAM, 9-point scale	none
UNBC-McMaster Pain Archive	2011	10.1109/fg.2011.5771462	Psychophysiologic al response dataset	Video	sensory intensity, affective-motivational, PSPI	Likert-type, PSPI	FACS

NIMH-ChEFS	2011	10.1002/mpr.343	Normed affective stimulus database	Faces	5 emotions, intensity	list selection, slider	none
UMB-DB	2011	10.1109/iccvw.2011.6130509	Dynamic/multimodal stimulus set	VR/AR	expressions, occlusions	not reported	3D acquisitions
D3DFACS	2011	10.1109/iccv.2011.6126510	Dynamic/multimodal stimulus set	Video	discrete emotions	not reported	FACS
Oulu-CASIA	2011	10.1016/j.imavis.2011.07.002	Dynamic/multimodal stimulus set	Video (NIR/Visible)	discrete emotions	not reported	none
ADFES	2011	10.1037/a0023853	Dynamic/multimodal stimulus set	Video	valence, arousal, discrete emotions	Likert	none
Ferré 380 set	2011	10.3758/s13428-011-0165-x	Normed affective stimulus database	Words	valence, arousal, concreteness	SAM/Likert	none
AGE	2011	10.1109/tsmcb.2010.2103557	Normed affective stimulus database	Words	valence, arousal, imagery	Likert	none
LANG Validation	2011	10.3758/s13428-010-0048-6	Normative adaptation/translation	Words	valence, arousal	SAM	fMRI/EEG
DENN-BAWL	2011	10.3758/s13428-011-0059-y	Normative adaptation/translation	Words	happiness, anger, fear, sadness	5-point Likert	none
Portuguese ANEW	2011	10.3758/s13428-011-0131-7	Normative adaptation/translation	Words	valence, arousal	SAM	none
GAPED	2011	10.3758/s13428-011-0064-1	Normed affective stimulus database	Images	valence, arousal, moral/legal norms congruence	0-100 scale	none

IAPS (Israeli Adaptation)	2011	10.1002/jts.20600	Normative adaptation/translation	Images	valence, arousal	SAM	none
EmoWisconsin	2011	10.1007/978-3-642-24571-8_7	Normed affective stimulus database	Speech	valence, activation, discrete emotions	SAM, categorical	none
MHi-Mimicry	2011	10.1007/978-3-642-24600-5_40	Psychophysiological response dataset	Multimodal	mimicry, affect	not reported	none reported
not specified	2011	10.1007/978-3-642-18184-9_35	Dynamic/multimodal stimulus set	Speech, Audio	not reported	not reported	none reported
Multi-PIE	2010	10.1016/j.imavis.2009.08.002	Emotion-recognition dataset	Faces	discrete emotions (smile, blink, etc)	discrete emotion ratings	none
CVRRCar-AVDB)	2010	10.1109/tmm.2010.2058095	CVRRCar-AVDB	Emotion-recognition dataset	Video; Multimodal; Speech	discrete emotions	discrete emotion ratings
not specified	2010	10.1007/978-3-642-12397-9_35	Dynamic/multimodal stimulus set	Audio, Video	6 basic emotions	subject agreement	acoustic features
Radboud Faces Database (RaFD)	2010	10.1080/02699930903485076	Normed affective stimulus database	Faces	8 expressions, intensity, clarity, genuineness, attractiveness, valence	forced-choice, 5-point scales	FACS
Extended Cohn-Kanade (CK+)	2010	10.1109/cvprw.2010.5543262	Psychophysiological response dataset	Video	7 emotions, FACS codes	FACS criteria	FACS
NVIE	2010	10.1109/tmm.2010.2060716	Dynamic/multimodal stimulus set	Multimodal	valence, arousal, discrete	3-point scale, 5-point scale	skin temperature

					emotions, intensity		
Multi-angle expression set	2010	10.1007/978-1-4419-6953-8_1	Psychophysiological response dataset	Video	not specified	not reported	FACS
FilmStim	2010	10.1080/02699930903274322	Normed affective stimulus database	Video	arousal, discrete emotions	PANAS, discrete emotion ratings	none
ANEW	2010	10.3758/brm.42.1.134	Normed affective stimulus database	Words	valence, arousal, dominance	SAM	none
ISAWS	2010	10.1007/s10508-010-9669-1	Domain-specific stimulus set	Words	sexual valence, discrete	SAM	Recognition
Self-IAPS (o set relevante para BPD)	2010	10.1521/pedi.2010.24.5.664	Domain-specific stimulus set	Images	valence, arousal, self-reference, ideal-other	Likert	none
not specified	2010	10.2466/pr0.106.2.581-588	Not applicable	Audio	self-report "elation"	7-point scale	none
Cam3D	2010	10.1109/tmm.2010.2052239	Dynamic/multimodal stimulus set	Multimodal, 3-D Faces	discrete emotions	Likert	none reported
not specified	2010	10.1007/978-3-642-12397-9_34	Dynamic/multimodal stimulus set	Audio	sentence modality	not reported	none reported
GENKI	2009	10.1109/tpami.2009.42	Dynamic/multimodal stimulus set	Video	4 emociones, intensidad	auto-reporte (0-5), juicio experto	none
STOIC	2009	10.1515/9783110216523.5.389	Dynamic/multimodal stimulus set	Multimodal	intensity for 7 emotions + neutral	continuous scroll bars	none

NimStim Set	2009	10.1016/j.psychres.2008.05.006	Normed affective stimulus database	Faces	8 expressions, proportion correct	semi-forced choice	none
UCDSEE	2009	10.1037/a0015766	Normed affective stimulus database	Faces	discrete emotions (self-conscious)	forced-choice	FACS
COST 2102	2009	10.3233/978-1-60750-072-8-51	Dynamic/multimodal stimulus set	Multimodal	discrete emotions	not reported	none
French Auditory Word Norms	2009	10.5334/pb-49-1-19	Normed affective stimulus database	Words	valence, arousal, threat	continuum ratings	none
IEMOCAP	2008	10.1007/s10579-008-9076-6	Dynamic/multimodal stimulus set	Multimodal, Mocap	discrete emotions, V/A/D	SAM, discrete labels	none
CAS-PEAL	2008	10.1109/tsmca.2007.909557	Emotion-recognition dataset	Faces	5 expressions	not reported	none
BU-4DFE	2008	10.1109/afgr.2008.4813324	Dynamic/multimodal stimulus set	Faces	discrete emotions	not reported	none
Bosphorus Database	2008	10.1007/978-3-540-89991-4_6	Dynamic/multimodal stimulus set	Video (3D)	discrete emotions	not reported	FACS
Eigenfaces Benchmark	2008	10.1109/icosp.2008.4697276	Emotion-recognition dataset	Faces	not specified	not reported	none
IAPS	2008	not reported	Normed affective stimulus database	Images	valence, arousal, dominance	SAM	none
VAM	2008	10.1109/icme.2008.4607572	Emotion-recognition dataset	Multimodal	valence, arousal, dominance	SAM	none

IADS Discrete Category Norms	2008	10.3758/brm.40.1.315	Normative adaptation/translation	Audio	discrete emotions	9-point scales	none
MULTIMOST	2008	10.1027/1618-3169.55.2.121	Dynamic/multimodal stimulus set	Multimodal	familiarity, valence, etc.	Likert	none
not specified	2007	10.1016/j.imavis.2005.12.021	Normed affective stimulus database	Video	discrete emotions, ground truth	discrete emotion ratings	none
Strauss & Allen set	2007	10.1080/02699930701319154	Normed affective stimulus database	Words	intensity, discrete cat	ratings/%	none
eNTERFACE'05	2006	10.1109/icdew.2006.145	Dynamic/multimodal stimulus set	Multimodal	6 archetypal emotions	expert judgment	none
BHU facial expression database	2006	10.1109/icmlc.2006.258460	Dynamic/multimodal stimulus set	Faces	not specified	not reported	none
FABO	2006	10.1109/icpr.2006.39	Emotion-recognition dataset	Faces	discrete emotions	discrete emotion ratings	none
Valence Pilot	2006	10.1016/j.paid.2006.03.025	Normed affective stimulus database	Words	valence	not reported	none
not specified	2005	10.1080/02699930541000084	Normative adaptation/translation	Video	discrete emotions	dimensional ratings	none
IAPS (Brazilian Adaptation)	2005	10.1590/s1516-44462005000300009	Normative adaptation/translation	Images	valence, arousal, dominance	SAM	none

Emo-DB	2005	10.21437/interspeech.2005-446	Normed affective stimulus database	Speech	discrete emotions, naturalness	Automated listening test	none
not specified	2005	10.1109/tpami.2005.90	Dynamic/multimodal stimulus set	Video, Faces	none reported	not reported	none reported
ASD	2004	10.1093/nar/gkh030	Domain-specific stimulus set	Speech	discrete emotions	discrete emotion ratings	none
not specified	2004	10.1007/978-3-540-24837-8_10	Normed affective stimulus database	Video	discrete emotions	discrete emotion ratings	none
SFEW	2004	10.1109/tsmcb.2004.825931	Normed affective stimulus database	Images	6 basic expressions + neutral	independent labellers	none
AR Face Database	2004	10.1037/t74376-000	Emotion-recognition dataset	Faces	expressions, illumination	not reported	none
Nevis'22	2003	10.1093/tandt/9.6.109	Emotion-recognition dataset	Images	classification categories	not reported	none
3-dimensional facial expressions set	2002	10.1016/s0165-0270(02)00006-7	Normed affective stimulus database	Faces	discrete emotions, intensity	Likert	none
Magdeburger Prosodie Korpus	2002	10.21437/speechprosody.2002-160	Normed affective stimulus database	Speech	prosodic identification	Questionnaire	none
IADS (Original)	2000	10.1111/1469-8986.3720204	Normed affective stimulus database	Audio	valence, arousal, dominance	SAM	EDA/GSR, ECG, EMG
Environmental Sounds Set	2000	10.1076/jcen.22.6.830.949	Normed affective stimulus database	Audio	naming accuracy,	Likert	none

					familiarity, pleasantness		
CMU PIE	1999	10.1016/s0168-9525(99)01806-5	Emotion-recognition dataset	Faces	neutral, smile, blink, talk	not reported	none
ANEW	1997	not reported	Normed affective stimulus database	Words	valence, arousal, dominance	SAM	none
DES	1997	10.21437/eurospeech.1997-482	Normed affective stimulus database	Speech	discrete emotions	Identification	none
ADSIP database	1995	10.1016/s0921-2647(06)80191-7	Dynamic/multimodal stimulus set	VR/AR	7 expressions, intensity	confidence ratings	none
not specified	1995	10.1080/02699939508408966	Normed affective stimulus database	Video	discrete emotions	Likert	none
Homograph Norms	1992	10.1080/02699939208411058	Domain-specific stimulus set	Words	threat value	associative	none
Bellezza norms	1986	10.3758/bf03204403	Normed affective stimulus database	Words	pleasantness	Likert	none

Note: EEG = electroencephalography; ERP = event-related potentials; EDA = electrodermal activity; fMRI = functional magnetic resonance imaging; ECG = electrocardiography; GSR = galvanic skin response; PPG = photoplethysmography; Temp = body temperature; CPM descriptors = component process model descriptors; FACS = facial action coding system; SAM = self-assessment manikin; VR/AR = virtual reality/augmented reality.

Data Availability Statement: The supplementary classification matrix generated for this review is openly available in the Open Science Framework (OSF) as *Supplementary Table S1. Bibliographic dataset of standardized stimulus resources*. In the OSF repository, the dataset is provided as bibliographic_dataset_of_standardized_stimulus_v1.0_2026.xlsx and bibliographic_dataset_of_standardized_stimulus_v1.0_2026.csv. Version 1.0 corresponds to the classification matrix used in the present manuscript and includes the standardized stimulus resources and related datasets identified through the structured narrative mapping. The dataset reports, where available, information on contribution type, database status, resource type, stimulus modality, affective norms or ratings, rating scales, physiological or neural data, and accessibility. The repository is available at: https://doi.org/10.17605/OSF.IO/KMFBS.
